# Targeting TREM1 augments antitumor T cell immunity by inhibiting myeloid-derived suppressor cells and restraining anti–PD-1 resistance

**DOI:** 10.1172/JCI167951

**Published:** 2023-11-01

**Authors:** Ashwin Ajith, Kenza Mamouni, Daniel D. Horuzsko, Abu Musa, Amiran K. Dzutsev, Jennifer R. Fang, Ahmed Chadli, Xingguo Zhu, Iryna Lebedyeva, Giorgio Trinchieri, Anatolij Horuzsko

**Affiliations:** 1 Georgia Cancer Center, Medical College of Georgia, Augusta University, Augusta, Georgia, USA.; 2 Laboratory of Integrative Cancer Immunology, Center for Cancer Research, National Cancer Institute, National Institutes of Health, Bethesda, Maryland, USA.; 3 Department of Chemistry and Physics, Augusta University, Augusta, Georgia, USA.

**Keywords:** Oncology, Therapeutics, Cancer immunotherapy, Cellular immune response, Melanoma

## Abstract

The triggering receptor expressed on myeloid cell 1 (TREM1) plays a critical role in development of chronic inflammatory disorders and the inflamed tumor microenvironment (TME) associated with most solid tumors. We examined whether loss of TREM1 signaling can abrogate the immunosuppressive TME and enhance cancer immunity. To investigate the therapeutic potential of TREM1 in cancer, we used mice deficient in *Trem1* and developed a novel small molecule TREM1 inhibitor, VJDT. We demonstrated that genetic or pharmacological TREM1 silencing significantly delayed tumor growth in murine melanoma (B16F10) and fibrosarcoma (MCA205) models. Single-cell RNA-Seq combined with functional assays during TREM1 deficiency revealed decreased immunosuppressive capacity of myeloid-derived suppressor cells (MDSCs) accompanied by expansion in cytotoxic CD8^+^ T cells and increased PD-1 expression. Furthermore, TREM1 inhibition enhanced the antitumorigenic effect of anti-PD-1 treatment, in part, by limiting MDSC frequency and abrogating T cell exhaustion. In patient-derived melanoma xenograft tumors, treatment with VJDT downregulated key oncogenic signaling pathways involved in cell proliferation, migration, and survival. Our work highlights the role of TREM1 in cancer progression, both intrinsically expressed in cancer cells and extrinsically in the TME. Thus, targeting TREM1 to modify an immunosuppressive TME and improve efficacy of immune checkpoint therapy represents what we believe to be a promising therapeutic approach to cancer.

## Graphical abstract

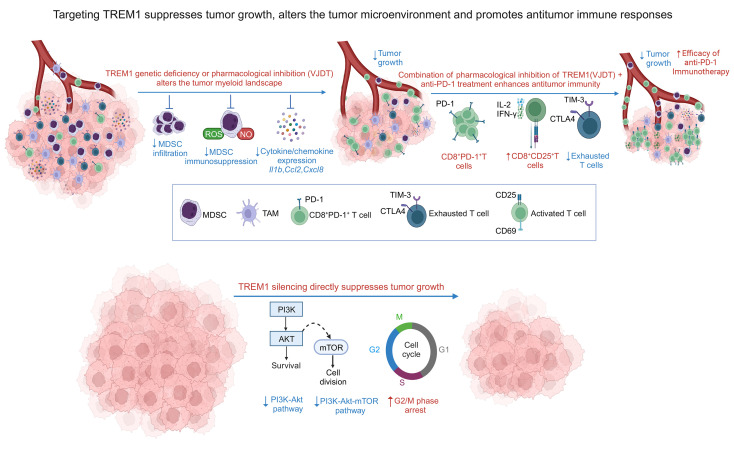

## Introduction

Triggering receptor expressed on myeloid cell 1 (TREM1) is a cell surface receptor and a member of the immunoglobulin superfamily that amplifies inflammatory responses by inducing the secretion of proinflammatory mediators (1, 2). TREM1 is mainly expressed on myeloid cells, such as neutrophils, monocytes/macrophages, and granulocytes (1, 3). Currently, the crucial pathophysiological role of TREM1 is defined not only in infectious diseases such as sepsis but also in atherosclerosis, ischemia reperfusion–induced tissue injury, colitis, fibrosis, and cancer (4, 5). Our group previously reported a TREM1-mediated mechanism of liver injury and fibrogenesis and described TREM1 as a master regulator of Kupffer cell activation, which escalates chronic liver inflammatory responses (6). Although the function of TREM1 in cancer is still unclear, TREM1 expression promotes tumorigenesis and supports tumor growth in various tumor models, including intestinal (7), pancreatic (8), and lung cancers (9) and hepatocellular carcinoma (10, 11). Several strategies have been developed to inhibit TREM1, including the use of biologics such as TREM1/Fc fusion proteins, monoclonal antibodies, and peptides (1, 3, 12–14). However, peptides are quickly degraded, causing a limited lifespan in the body. Thus, the development of TREM1-specific small molecule inhibitors is a more attractive approach. Recently, it was reported that the flavonoid morin hydrate (MH;2′,3,4′,5,7-pentahydroxyflavone) inhibits the TREM1/TLR4–mediated inflammatory response in murine macrophages and protects against acute liver injury (15). Since targeting both TREM1 and TLR4 is not advantageous in most clinical pathological conditions, the development of TREM1-specific small molecule inhibitors is an attractive strategy to control the TREM1 signaling pathway. Here, we have developed what we believe to be a novel TREM1 small molecule inhibitor to target the TREM1 signaling pathway.

In the tumor microenvironment (TME), protective cancer immunity is frequently hampered by suppressive immune cell populations, such as myeloid-derived suppressor cells (MDSCs), regulatory T (Treg) cells, and tumor-associated macrophages (TAMs), that favor immune escape (16, 17). Increasing evidence suggests that tumor-intrinsic signaling pathways play a crucial role in regulating the immunosuppressive TME (15, 18, 19). In patients with non-small cell lung cancer (NSCLC), TREM1 expression in TAMs correlates with tumor recurrence and poor survival (20). The expression of TREM1 and proinflammatory cytokine (TNF, IL-1β) were upregulated in blood monocytes cocultured with lung cancer cells from patients with NSCLC (20). More recently, TREM1 signaling in TAMs was shown to impair the antitumor activity of CD8^^+^^ T cells through the recruitment and activation of Tregs, thereby inducing resistance to anti-PD-L1 treatment (19). Overall, the results of these studies, along with our previous reports (5, 6), prompted us to investigate the role of TREM1 in the crosstalk between tumor cells and the immune microenvironment.

To investigate the therapeutic potential of TREM1 in cancer, we used mice deficient in *Trem1* or we treated WT mice with a novel small molecule TREM1 inhibitor, VJDT. We found that the growth of mouse melanoma (B16F10) and fibrosarcoma (MCA205) tumors was delayed in *Trem1^–/–^* mice or in *Trem1^+/+^* mice treated with VJDT. Single-cell RNA-Seq (scRNA-Seq) combined with functional assays of *Trem1*^^–/–^^ tumor infiltrates revealed, in the absence of TREM1 signaling, a decreased immunosuppressive capacity of MDSCs and increased PD-1 expression on CD8^^+^^ T cells. In vivo inhibition of TREM1 with VJDT synergized with anti-PD-1 treatment by limiting MDSC frequency. Melanoma patient-derived xenograft (PDX) tumors treated with VJDT downregulated key oncogenic signaling pathways involved in cell proliferation, migration, and survival. In cohorts of patients with liver hepatocellular carcinoma (LIHC) and glioblastoma multiform (GBM), high TREM1 expression was associated with worse overall survival and positively correlated with immune trafficking and myeloid cell recruitment. Our findings reveal a role of TREM1 in promoting tumor-intrinsic oncogenic pathways and MDSC tumor-infiltration, thus contributing strongly to an immunosuppressive state. Therefore, blockade of TREM1 signaling may constitute an attractive novel and double-hit approach for improving current immunotherapies.

## Results

### TREM1 deficiency and antagonism restrain tumor growth by modifying tumor immune infiltrates.

**Figure 1:**
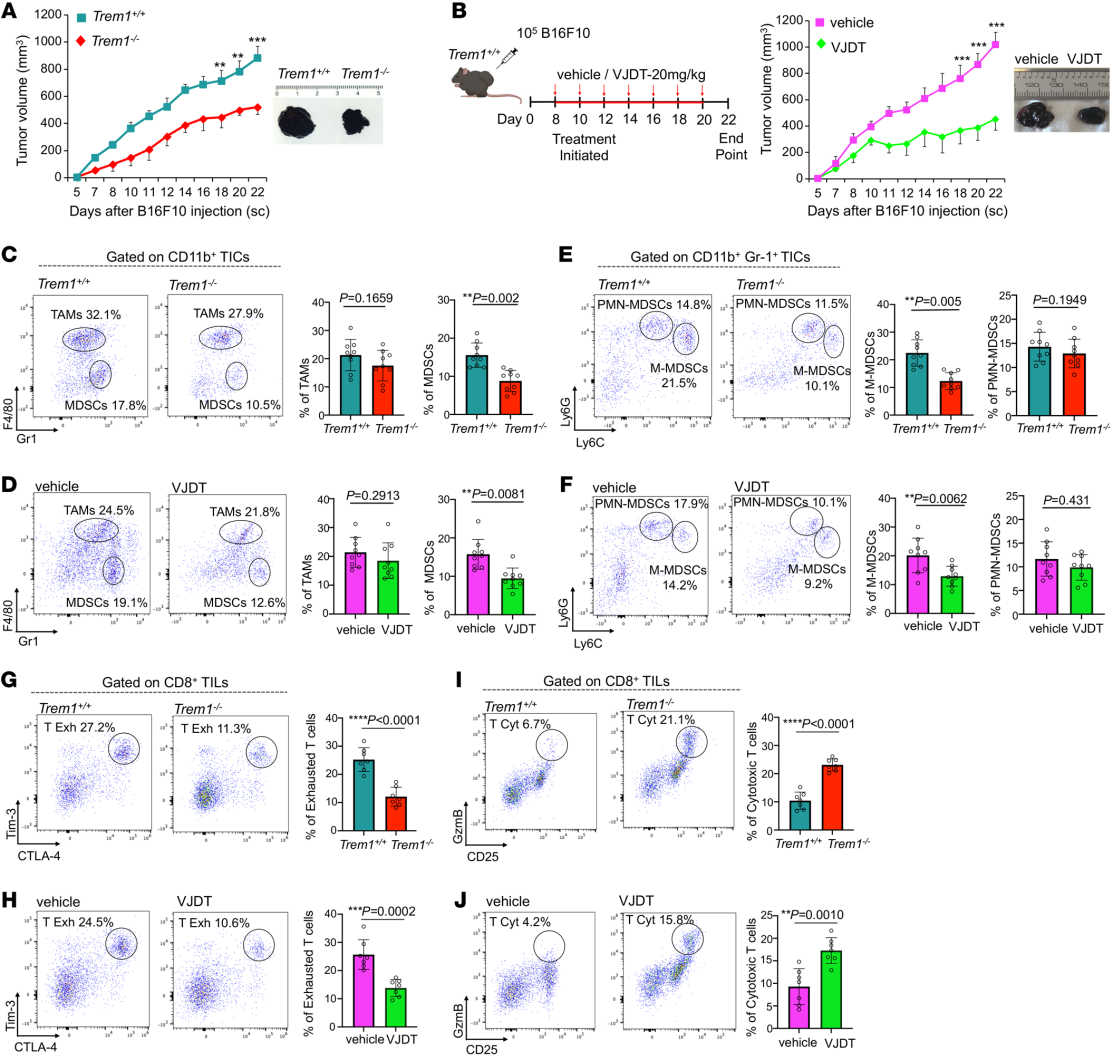
TREM1 deficiency and anti-TREM1 treatment diminish B16F10 tumor growth by altering tumor immune infiltrates. (**A**) Tumor growth curves for B16F10 melanoma in *Trem1^+/+^* and *Trem1^–/–^* mice (mean ± SEM, *n* = 9 mice/group). Representative microscopic images of tumors from indicated groups on day 22. (**B**) Schematic illustration describes treatment protocol with TREM1 inhibitor VJDT on B16F10 melanoma in *Trem1^+/+^* mice. Treatment initiated on eighth day of tumor growth followed every alternate day with VJDT (20 mg/kg) or vehicle (DMSO) until day 20. Tumor growth curves calculated by individual measurements recorded every alternate day (*n* = 9 mice/group, mean ± SEM). Representative microscopic images of tumors from indicated groups on day 22. (**C**–**J**) Tumors harvested on day 22. Flow cytometry analysis and frequency of cells in gated immune subsets are depicted (dot plots show a representative experiment performed in triplicate, *n* = 7–9 mice/group, mean ± SD shown). (**C**) Frequency of CD11b^+^F4/80^+^Gr-1^–^ TAMs and CD11b^+^F4/80^–^Gr-1^+^ MDSC infiltrates in *Trem1^+/+^* or *Trem1^–/–^* mice and (**D**) in *Trem1^+/+^* mice with indicated treatment. (**E**) Frequency of CD11b^+^Gr-1^+^Ly6C^hi^Ly6G^–^ M-MDSC and CD11b^+^Gr1^+^Ly6C^lo^Ly6G^+^ PMN-MDSCs in *Trem1^+/+^* or *Trem1^–/–^* mice and (**F**) in *Trem1^+/+^* mice with indicated treatment. (**G**) Frequency of exhausted CD8^+^Tim-3^+^CTLA-4^+^ T cells in *Trem1^+/+^* or *Trem1^–/–^* mice and (**H**) in *Trem1^+/+^* mice with indicated treatment. (**I**) Frequency of cytotoxic CD8^+^GzmB^+^CD25^+^ T cells in *Trem1^+/+^* or *Trem1^–/–^* mice and (**J**) in *Trem1^+/+^* with indicated treatment. **P < 0.01; ***P < 0.001; ****P < 0.0001 assessed by 2-way ANOVA for multiple comparison of longitudinal tumor growth between groups (**A** and **B** [tumor growth]) or 2-tailed Student’s *t* test for comparison between 2 groups (**C**–**H**).

**Figure 2:**
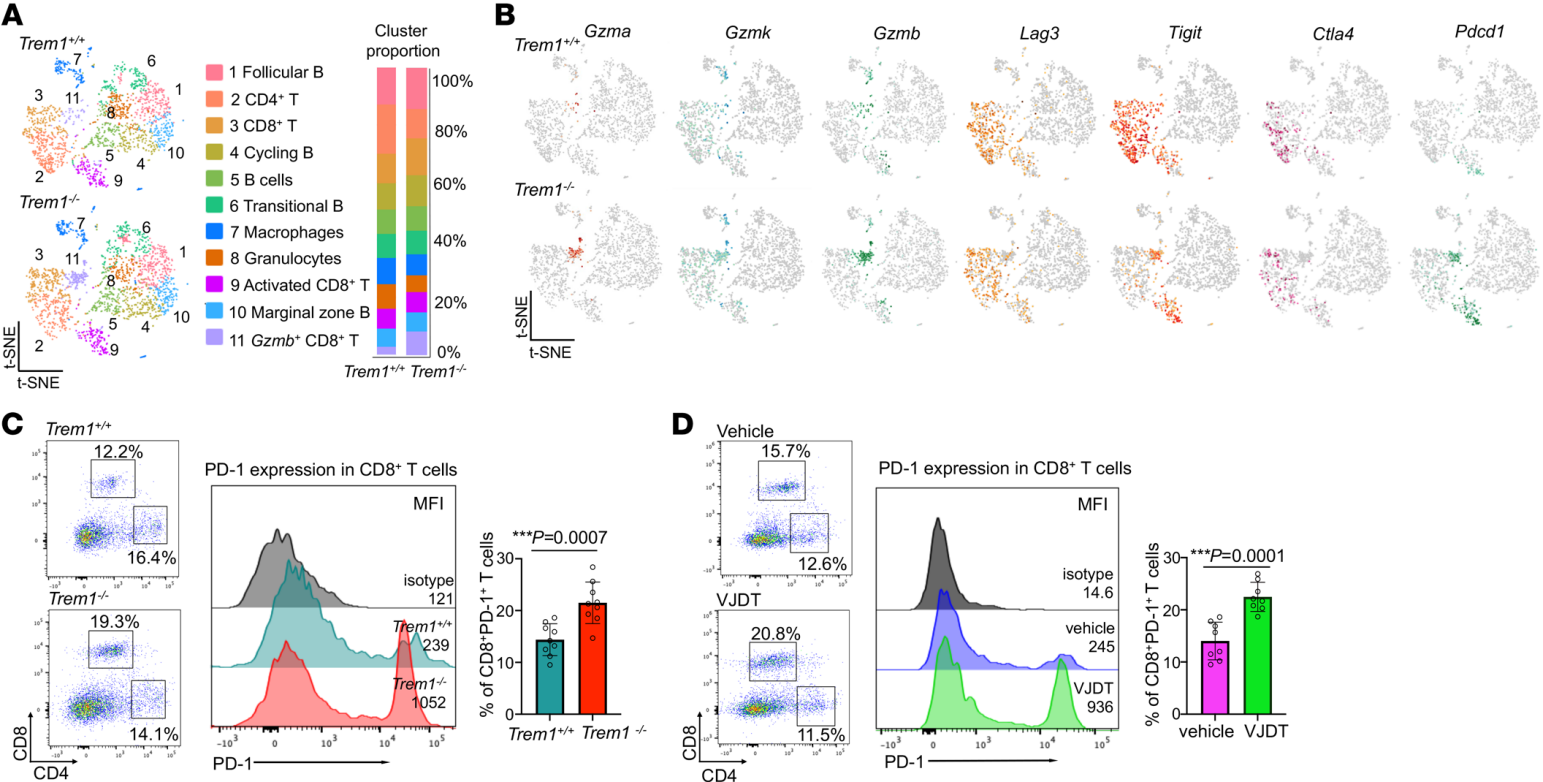
scRNA-Seq analysis reveals alterations in immune landscape of TREM1-deficient TME in B16F10 melanoma. scRNA-Seq analysis of tumor-infiltrating CD45^+^ cells from melanoma-bearing *Trem1^+/+^* and *Trem1^–/–^* mice at day 22. For each experimental group, 4 biological replicates were pooled. (**A**) Data analyzed by Loupe browser and Seurat to generate t-SNE plot depicting differential cell clusters and their frequencies. Cluster identities based on expression of key gene signatures described in Methods. Bar graphs depict cluster proportions in each condition (*Trem1^+/+^* and *Trem1^–/–^*). (**B**) t-SNE plots characterize expression of specific cluster markers for TICs in *Trem1^+/+^* and *Trem1^–/–^* tumor-bearing mice. (**C**) Flow cytometry histogram plots depict PD-1 expression in tumor-infiltrating CD8^+^ and CD4^+^ T cells of *Trem1^+/+^* or *Trem1^–/–^* mice and (**D**) in *Trem1^+/+^* mice with indicated treatment (dot plots show a representative experiment performed in triplicate, *n* = 8–9 mice/group, mean ± SD). ***P < 0.001 assessed by 2-tailed Student’s *t* test for comparison between 2 groups (**C** and **D**).

To analyze the impact of TREM1 on tumor growth and immune responses, we used 2 transplantable syngeneic mouse tumor models, the B16F10 melanoma and MCA 205 fibrosarcoma cell lines, known to be associated with a microenvironment infiltrated with myeloid and lymphoid cells (21, 22). In *Trem1*^^+/+^^ mice, these tumors grew rapidly, but their growth was significantly attenuated in *Trem1*^^–/–^^ mice (Figure 1A and Supplemental Figure 1A; supplemental material available online with this article; https://doi.org/10.1172/JCI167951DS1). To demonstrate the therapeutic benefit of pharmacological inhibition of TREM1, we developed VJDT, a novel TREM1 small molecule inhibitor that effectively blocks TREM1 signaling (VJDT design and development and toxicity studies are described in Methods and Supplemental Figure 2, A–L). VJDT treatment significantly delayed the growth of both tumor types (Figure 1B and Supplemental Figure 1B). We next examined the effect of TREM1 deficiency and its antagonism by VJDT on the tumor immune infiltrates. Within the myeloid compartment, lack of TREM1 signaling reduced the number of TAMs (CD11b^^+^^F4/80^^+^^Gr1^^–^^) (23) while significantly decreasing the proportion of MDSCs (CD11b^^+^^F4/80^^–^^Gr1^^+^^) (Figure 1, C and D, Supplemental Figure 1, C and D, and Supplemental Figure 3A). Furthermore, within the MDSC population, only the monocytic MDSCs (M-MDSCs; CD11b^^+^^F4/80^^–^^Gr1^^+^^Ly6C^^hi^^Ly6G^^–^^) but not the polymorphonuclear MDSCs (PMN-MDSCs; CD11b^^+^^F4/80^^–^^Gr1^^+^^Ly6C^^lo^^Ly6G^^+^^) (24, 25) were attenuated by TREM1 antagonism (Figure 1, E and F, Supplemental Figure 1, E and F, and Supplemental Figure 3B). In addition, among the tumor-infiltrating lymphocytes (TILs), TREM1 deficiency led to significant attenuation in exhausted CD8^^+^^ T (T Exh) cells (CD8^^+^^Tim-3^^+^^CTLA-4^^+^^) (26) (Figure 1, G and H, Supplemental Figure 1, G and H, and Supplemental Figure 3C) while increasing the proportion of cytotoxic CD8^^+^^ T (T Cyt) cells (CD8^^+^^GzmB^^+^^CD25^^+^^) (27) (Figure 1, I and J, Supplemental Figure 1, I and J, and Supplemental Figure 3D). CD3^^+^^CD4^^+^^ T cells were equally represented in all groups; in contrast, CD8^^+^^ T cells were selectively enriched within the TREM1-deficient TME. To gain a global understanding of the impact of TREM1 silencing, we analyzed the CD45^^+^^ tumor-infiltrating cells (TICs) of B16F10 tumor-bearing *Trem1*^^+/+^^ and *Trem1*^^–/–^^ mice. Utilizing the 10× genomics chromium platform, we analyzed approximately 5,390 cells per sample with a coverage rate of 15,493 genes per cell. Dimensional reduction by t-distributed stochastic neighbor embedding (t-SNE) identified 11 distinct clusters of TICs within the melanoma TME (Figure 2A). These clusters were classified based on expression of canonical gene markers from a comparative data set in the Immunological Genome Project (ImmGen) Consortium. TREM1 silencing led to expansion of Granzyme B-expressing CD8^^+^^ T cells in Cluster 11 while decreasing the frequency of CD4^^+^^ T cells in Cluster 2 within the TME (Figure 2A). Furthermore, genes associated with T cell cytotoxicity, such as *Gzma, Gzmk,* and *Gzmb* (28), showed increased expression; in contrast, markers of T cell exhaustion, including *Lag3*, *Tigit,* and *Ctla4* (29), were diminished within the *Trem1*^^–/–^^ TME (Figure 2B). Interestingly, in scRNA-Seq analysis, one of the T cell markers, *Pdcd1* (29, 30), was elevated during TREM1 silencing, and our flow cytometry data further confirmed this observation, wherein TREM1 deficiency or VJDT treatment induced significant increases in tumor-infiltrating CD8^^+^^PD-1^^+^^ T cells within the TME (Figure 2, C and D and Supplemental Figure 1, K and L). As assessed by scRNA-Seq analysis, other TIC populations, such as B cells, were not affected by TREM1 silencing, while macrophages and granulocytic cells were chronically under-represented in this experiment for comparative analysis. However, supporting data from flow cytometry and scRNA-Seq analysis suggested that TREM1 deficiency globally remodels the TME toward a uniquely more immunopermissive state by diminishing M-MDSC infiltration and CD8^^+^^ T cell exhaustion while promoting CD8^^+^^ T cell infiltration. Further, our data shows augmented *Pdcd1* expression on CD8^^+^^ T cells within the *Trem1*^^–/–^^ TME, suggesting that pharmacological inhibition of TREM1 could be a potential therapeutic strategy to overcome resistance in PD-1 immune checkpoint therapy while boosting antitumor immune responses.

### TREM1 deficiency alters the tumor myeloid landscape.

**Figure 3:**
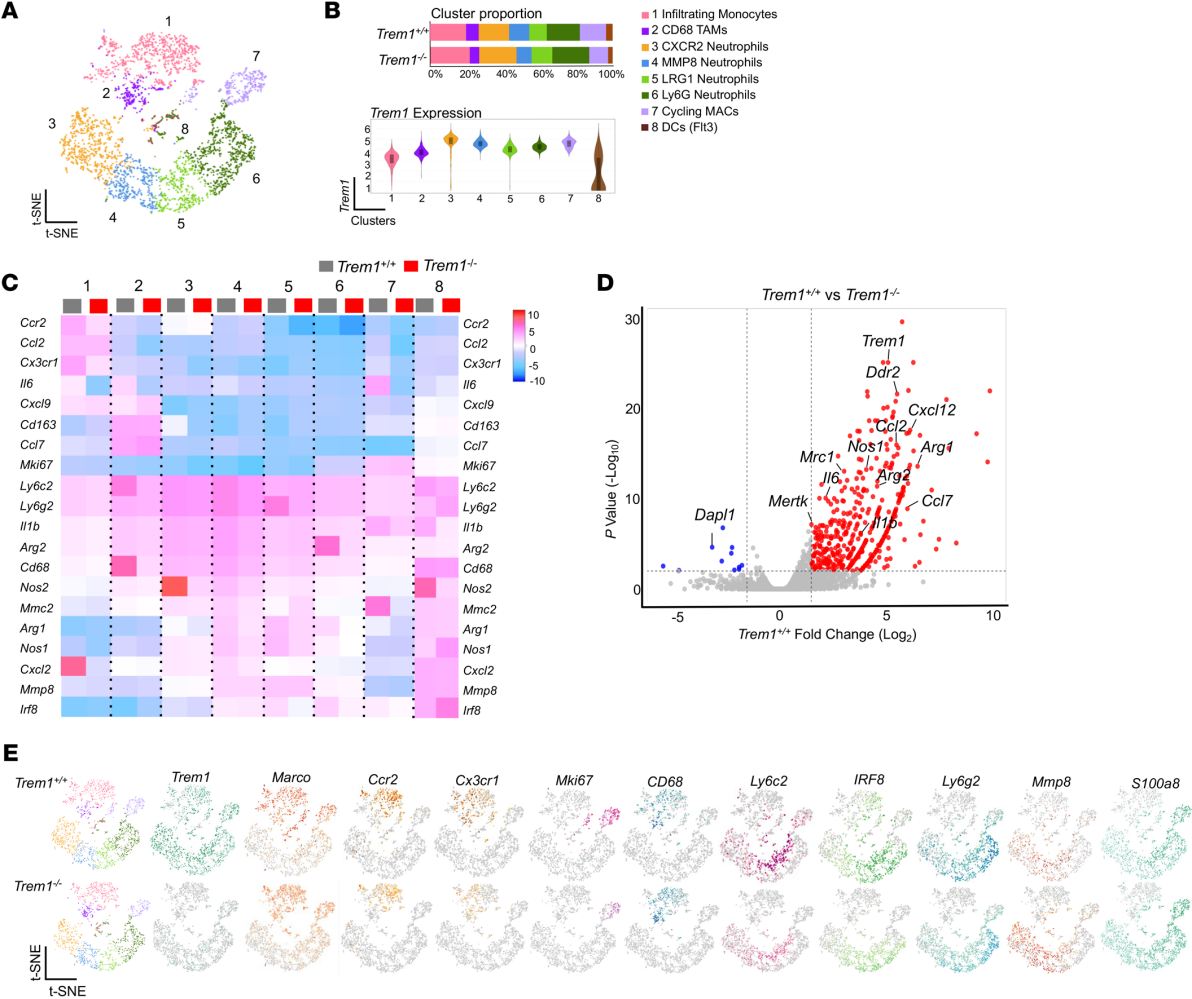
TREM1 deficiency alters the myeloid landscape in B16F10 tumors. scRNA-Seq analysis of tumor-infiltrating CD45^+^CD11b^+^ cells from melanoma-bearing *Trem1^+/+^* and *Trem1^–/–^* mice at day 22. For each experimental group, 5 biological replicates were pooled. (**A**) Data analyzed by Loupe browser and Seurat to generate t-SNE plot depicting differential cell clusters and their frequencies within tumor myeloid populations from merged conditions. Clusters classified based on expression of key genes described in Methods. (**B**) Bar graphs represent cluster proportions in each condition (*Trem1^+/+^* and *Trem1^–/–^*). Violin plots characterize *Trem1* expression among different clusters in *Trem1^+/+^* mice. (**C**) Heatmap shows expression of selected genes of interest in each myeloid cluster for *Trem1^+/+^* and *Trem1^–/–^* tumor-bearing mice. (**D**) Volcano plot shows differentially expressed genes of *Trem1^+/+^* TICs compared with *Trem1^–/–^*. Red dots represent upregulated genes with fold change greater than 1.5 and *P* < 0.05; blue dots show downregulated genes. Y axis denotes -Log_10_ *P,* while X axis shows Log_2_ fold change. (**E**) t-SNE plots characterize expression profile of cluster markers in TICs from *Trem1^+/+^* and *Trem1^–/–^* tumor-bearing mice.

**Figure 4:**
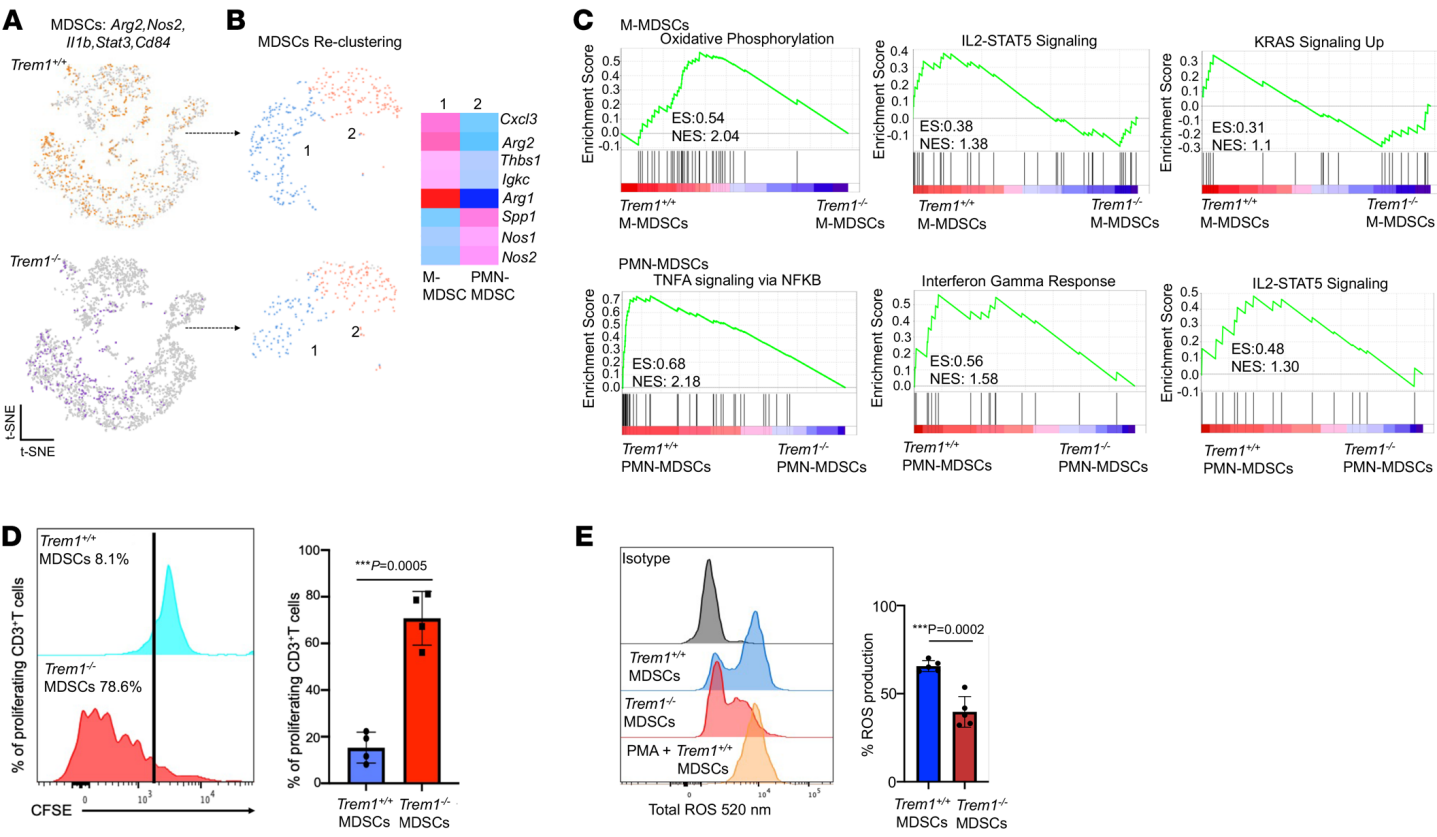
TREM1 deficiency restricts immunosuppressive capacity of MDSCs in B16F10 melanoma. (**A**) t-SNE plots describe distribution of *Arg2, Nos2*, *Il1b, Stat3,* and *Cd84*–expressing tumor-infiltrating MDSCs in melanoma-bearing *Trem1^+/+^* and *Trem1^–/–^* mice. For each experimental group, 5 biological replicates were pooled. (**B**) t-SNE plots describe reclustering analysis of MDSCs into 2 subsets. Heatmap depicts expression profile of specific genes in the M-MDSC and PMN-MDSC clusters. (**C**) GSEA depicts enrichment of hallmark pathways in tumor-infiltrating M-MDSCs and PMN-MDSCs of *Trem1^+/+^* mice compared with *Trem1^–/–^*. Top enriched pathways shown with enrichment score (ES) and normalized enrichment score (NES). (**D**) MDSC suppression assay using CFSE-labeled T cells from *Trem1^+/+^* mice primed with anti-CD3 and anti-CD28, cocultured with CD11b^+^Gr-1^+^ MDSCs from *Trem1^+/+^* (blue) or *Trem1^–/–^* (red) tumor-bearing mice. (**E**) ROS formation assay of CD11b^+^Gr-1^+^ MDSCs from *Trem1^+/+^* (blue) or *Trem1^–/–^* (red) tumor-bearing mice (*n* = 5 mice/group, mean ± SD), assessed by flow cytometry. ***P < 0.001 assessed by 2-tailed Student’s *t* test for comparison between 2 groups (**D** and **E**).

Our scRNA-Seq analysis of CD45^^+^^ TICs in the melanoma model demonstrated a substantial alteration of the tumor immune landscape under *Trem1* silencing. Additionally, to comprehensively characterize the impact of TREM1 deficiency specifically within the tumor myeloid populations, we selectively enriched the CD45^^+^^CD11b^^+^^ tumor–infiltrating myeloid cells from tumor-bearing *Trem1*^^+/+^^ and *Trem1*^^–/–^^ mice for scRNA-Seq analysis. We assayed up to 4,200 isolated CD45^^+^^CD11b^^+^^ cells from the TME of the B16F10 melanoma model with a coverage rate of 18,854 genes per cell. t-SNE analysis resolved 8 distinct cell clusters of tumor-infiltrating myeloid cell types (Figure 3A). The different subsets of macrophages and granulocytic populations within the tumor infiltrates were annotated using the expression of canonical gene markers derived from a comparable data set from the ImmGen Consortium. Macrophage classical markers such as *Marco* and *Ly6c2* (31, 32) were higher in clusters 1, 2, 5, and 7 (Figure 3, B and E). Cluster 1 exhibited characteristics of infiltrating monocytes expressing monocytic markers such as *Ccr2* and *Cx3cr1*, indicating that these cells had recently migrated from the blood (32), while cluster 2 was defined as TAMs that were positive for *Cd68* and *Cd163* (33, 34) (Figure 3, B and E). Cluster 7 of the macrophage subset exhibited *Mki67,* a unique marker for the cell cycle, and was defined as cycling macrophages (8, 32) (Figure 3, B and E). Clusters 3, 4,5, and 6 were categorized as neutrophils due to their relatively higher expression of *Ly6g2*, *S100a8,* and *MMP8* (35). Cluster 8 was identified as dendritic cells, due to elevated expression of *Flt3,* which was poorly represented in both groups. Our data demonstrated that TREM1 deficiency was associated with decreased frequency of tumor-infiltrating *Ccr2* and *Cx3cr1* monocytes of cluster 1 (Figure 3, B and E). Their decrease in *Trem1*^^–/–^^ mice suggests an attenuation of the cytokine/chemokine signaling network essential for tumor infiltration. In addition, *Mki67* cycling macrophages that expressed high levels of *Trem1* were dramatically diminished in the *Trem1*^^–/–^^ group, indicating the indispensable role of TREM1 in survival and maintenance of these cells (Figure 3B). Interestingly, TREM1 deficiency led to an increase in *Cxcr2*-positive infiltrating neutrophils (Cluster 3) as well as the *Lrg1*-expressing activated neutrophils (Cluster 5), while diminishing the frequency of *MMP8*-expressing granulocytic neutrophils. These considerable changes in the neutrophil clusters during *Trem1* silencing are indicative of their integral role in neutrophil recruitment and activation (Figure 3B). A heatmap of the normalized expression of selected genes in each cluster showed that expression of *Ccr2* and *Cx3cr1,* which encode the major chemokine receptors involved in macrophage recruitment (36, 37), along with their ligands *Ccl2* and *Cxcl2* (38, 39), were downregulated in the *Trem1^–/–^* B16F10 tumors (Figure 3C). Moreover, proinflammatory cytokine transcripts such as *Il1b, Ccl7, Cxcl12,* and *Il6* (40) and genes associated with immunosuppression, including *Mrc1*, *Arg1,* and *Mertk* (41, 42), were substantially elevated in all clusters within the *Trem1*^^+/+^^ tumors compared with the *Trem1*^^–/–^^ tumors (Figure 3D). These data, together with flow cytometry analysis (Figure 1, C and E), suggest that the tumor-infiltrating myeloid cell landscape is altered in the TREM1-deficient TME. Overall, our findings suggest that TREM1 deficiency leads to a considerable reconditioning of the tumor-myeloid landscape by inhibiting macrophage recruitment. Next, we characterized the impact of TREM1 deficiency on MDSC populations within tumor infiltrates. We defined MDSCs as *Arg2, Nos2*, *Il1b, Stat3,* and *Cd84–*expressing cells (43–45) that were diffusely represented in various clusters as they could not be categorized separately as a distinct cell population (Figure 4A). Reclustering analysis of MDSCs revealed that these cells were composed of 2 distinct clusters, clusters 1 and 2. A comparative expression profile of specific markers between the 2 groups revealed that Cluster 1 can be categorized as M-MDSCs due to elevated expression of *Cxcl3*, *Arg1*, and *Arg2*, while Cluster 2 is classified as PMN-MDSCs due to elevated expression in *Nos1, Nos2, and Spp1* (Figure 4B). A reduced infiltration of M-MDSCs was determined in the TME of TREM1-deficient mice (Figure 4, A and B). Moreover, Gene Set Enrichment Analysis (GSEA) revealed that TREM1 deficiency in M-MDSCs attenuated the oxidative phosphorylation and IL-2 signaling pathways, which have been associated with both MDSC survival and T cell suppressor activity (46, 47). In addition, in PMN-MDSCs, *Trem1* silencing inhibited the IFN-γ and TNF signaling pathways (Figure 4C). Our observations hypothesize a role of TREM1 in controlling the inhibitory capacity of these immunosuppressive cells. To test this hypothesis, we compared the immunosuppressive capacity of MDSCs isolated from *Trem1*^^+/+^^ and *Trem1*^^–/–^^ tumor-bearing mice. Tumor-infiltrating MDSCs harvested from TREM1-deficient mice exhibited diminished suppressive capacity on the proliferation of CD3^^+^^ T cells activated with anti-CD3/anti-CD28 compared with their TREM1-positive counterparts (Figure 4D). Furthermore, ROS formation assays showed that MDSCs isolated from *Trem1*^^–/–^^ tumor-bearing mice had significantly decreased ROS formation capabilities compared with *Trem1*^^+/+^^ MDSCs (Figure 4E). Collectively, these data suggested that TREM1 deficiency limits tumor progression by reconditioning the myeloid infiltrate in the TME via reduced accumulation and immunosuppressive capacity of macrophages and MDSCs.

### TREM1 inhibition augments anti-PD-1 immunotherapy.

**Figure 5:**
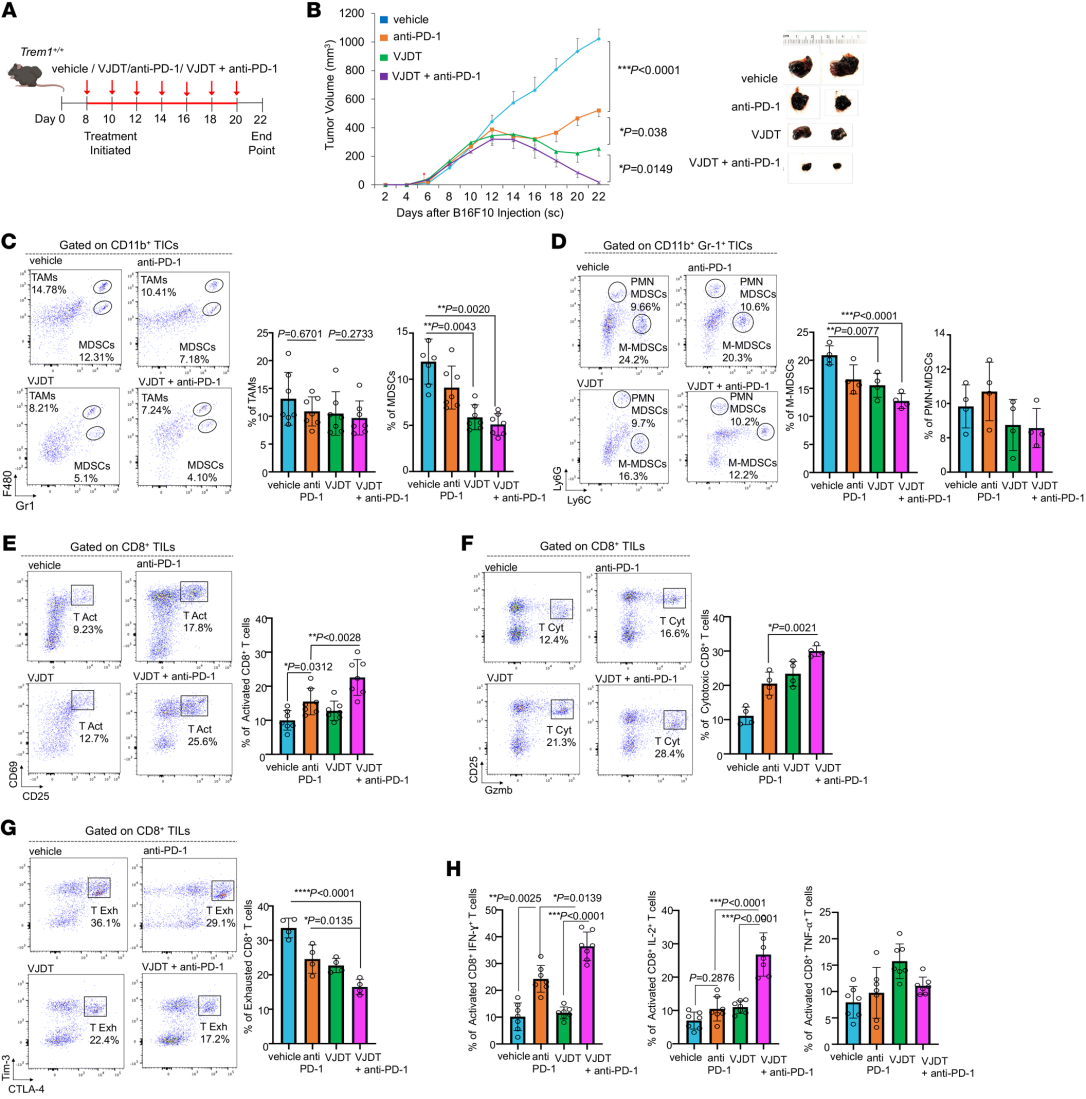
TREM1 inhibition enhances anti-PD-1 response by attenuating MDSC frequency and augments CD8^+^T cell immunity. (**A**) Schematic illustrates various treatment regimens on B16F10 tumors in *Trem1^+/+^* mice. Melanoma-bearing *Trem1^+/+^* mice were treated with either vehicle (combination of DMSO and IgG2a Isotype) or 200 μg anti-PD-1 antibody or 20 mg/kg VJDT or a combination of both from day 8 until day 20, on every alternate day of tumor progression. (**B**) Tumor growth curves expressed as overall tumor volume monitored every alternate day (*n* = 7 mice/group, mean ± SEM). Representative microscopic images of tumors from the indicated groups on day 22 are shown. (**C**–**H**) Tumors were harvested on day 22. Flow cytometry analysis of gated immune subset cells are shown (plots depict 1 representative experiment performed in triplicate, *n* = 4–7mice/group, mean ± SD). (**C**) Frequency of CD11b^+^F4/80^+^Gr-1^–^ TAMs, CD11b^+^F4/80^–^Gr-1^+^ MDSCs, and (**D**) CD11b^+^Gr-1^+^Ly6C^hi^Ly6G^–^ M-MDSCs and CD11b^+^ Gr1^+^Ly6C^lo^Ly6G^+^ PMN-MDSCs in the TME of the indicated groups. (**E**) Frequency of activated CD8^+^CD69^+^CD25^+^ T cells, (**F**) cytotoxic CD8^+^GzmB^+^CD25^+^ T cells, and (**G**) exhausted CD8^+^Tim-3^+^CTLA-4^+^ T cells within the TME. (**H**) Frequency of tumor-infiltrating CD8^+^CD25^+^ T cells expressing either IFN-γ, IL-2, or TNF-α. **P < 0.01; ***P < 0.001; ****P < 0.0001 by 2-way ANOVA for multiple comparison of longitudinal tumor growth between groups (**B** [tumor growth]) or 2-tailed Student’s *t* test for comparison between 2 groups (**C**–**H**).

**Figure 6:**
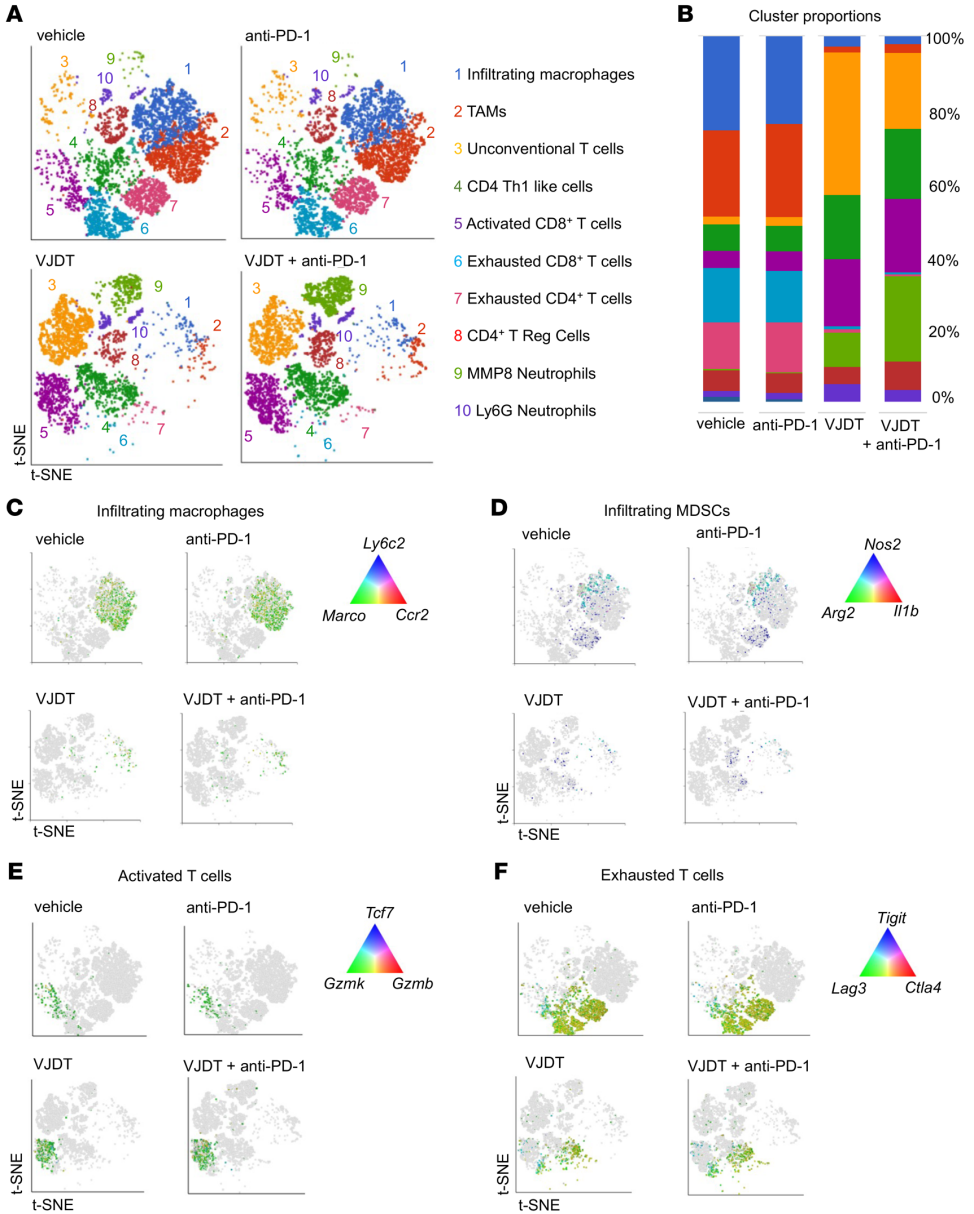
scRNA-Seq analysis reveals alterations of TICs in TREM1 inhibition with anti-PD-1 treatment of B16F10 melanoma. scRNA-Seq of tumor-infiltrating CD45^+^ immune cells sorted from TME of B16F10 melanoma-bearing *Trem1^+/+^* mice receiving either VJDT, anti-PD-1, both in combinational treatment, or vehicle. For each experimental group, 3 biological replicates were pooled. (**A**) scRNA-Seq data analyzed using Partek Flow to generate t-SNE plot showing differential cell clusters and their frequencies within the TME. Cluster identities were annotated based on expression of key gene signatures as described in Methods. (**B**) Bar graph depicts cluster proportions associated with each treatment group. (**C**–**F**) t-SNE plots characterize changes during treatment in the expression profile of key genes involved in (**C**) infiltrating macrophages, (**D**) infiltrating MDSCs, (**E**) activated T cells, and (**F**) exhausted T cells across the indicated groups.

**Figure 7:**
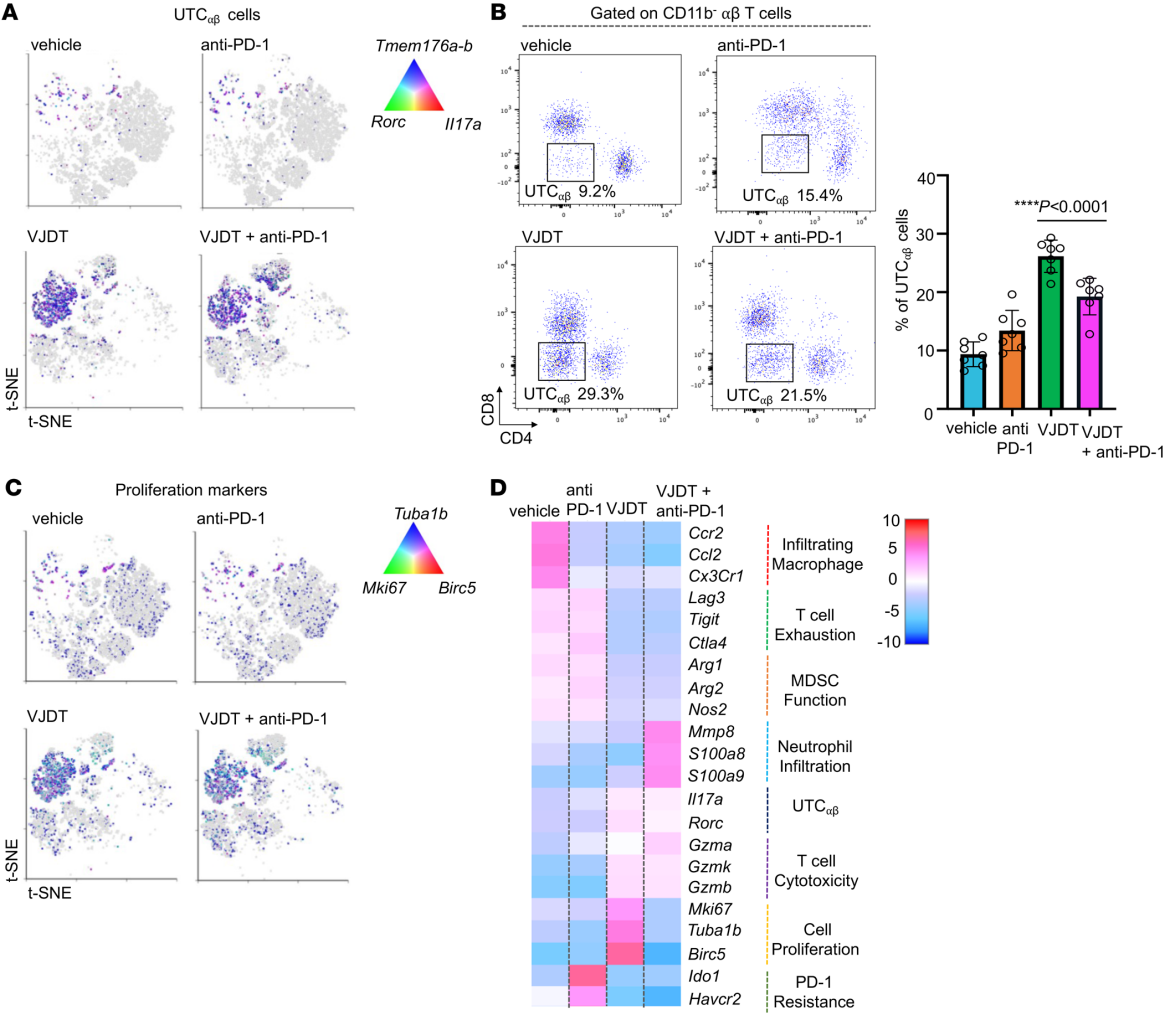
scRNA-Seq analysis of tumor infiltrating UTC_αβ_ cells in TREM1-inhibited TME of B16F10 melanoma. (**A**) t-SNE plot describes expression profile of key gene markers for UTC_αβ_ cells in melanoma-bearing *Trem1^+/+^* mice receiving either VJDT, anti-PD-1, both in combinational treatment, or vehicle. For each experimental group, 3 biological replicates were pooled. (**B**) Flow cytometry analysis shows frequency of UTC_αβ_ cells within the TME of *Trem1^+/+^* mice across indicated groups (*n* = 7 mice/group, mean ± SD). (**C**) t-SNE plot describes global expression profile of proliferation markers across indicated groups. (**D**) Heatmap depicts differential transcription profiles of tumor-infiltrating CD45^+^ immune cells sorted from TME of indicated groups. The differentially expressed genes associated with effector function are shown. ****P < 0.0001 by 1-way ANOVA using Dunnett’s multiple comparison test (**B**).

Our flow cytometry and scRNA-Seq data demonstrated that TREM1 deficiency was strongly associated with an increase in *Pdcd1* expression and expansion of CD8^^+^^PD-1^^+^^ T cells within the TME (Figure 2, C and D). Low expression of PD-1 and/ or decreased frequency of CD8^^+^^ PD-1^^+^^ T cells are biomarkers for resistance to PD-1 immune checkpoint blockade (ICB) therapy in cancer patients (48). Therefore, we hypothesized that combining TREM1 inhibition by VJDT and a PD-1 inhibitor would improve the overall efficacy of PD-1-based ICB therapy. To demonstrate this, *Trem1^+/+^* mice with B16F10 melanoma were treated with either anti-PD-1, VJDT, or a combination of both (Figure 5A). Monotherapy with either of the inhibitors alone only delayed tumor growth, while the combinational treatment with both anti-PD-1 and VJDT led to a significant reduction in overall tumor growth (Figure 5B). Flow cytometry analysis of the TICs in the various treatment groups showed that VJDT treatment induced significant attenuation in MDSC frequency with minimal effect on TAMs (Figure 5C). Moreover, the combinational treatment restricted M-MDSC populations more efficiently than monotherapy with VJDT (Figure 5D). Clinical efficacy of ICB therapies is reflected on the immunological state of CD8^^+^^ T cells within the TME (49). Herein, the combinational regimen led to significant expansion in activated CD8^^+^^ T (T Act) cells (CD8^^+^^CD69^^+^^CD25^^+^^ T cells) (50) (Figure 5E) as well as cytotoxic CD8^^+^^ T cells (Figure 5F) compared with the other therapies. In contrast, exhausted CD8^^+^^ T cells were significantly reduced in combinational treatment even more effectively than anti-PD-1 monotherapy (Figure 5G). In line with the cell populations, cytokine analysis revealed that combinational treatment also increased the population of tumor-infiltrating T cells producing IL-2 and IFN-γ (but not TNF-α) significantly more than anti-PD-1 blockade alone (Figure 5H). Increased production of IFN-γ is a marker for the transition from a T exhaustive phenotype to a T effector cell phenotype (51, 52). Furthermore, to comprehensively characterize the changes within the TME during TREM1 deficiency and anti-PD-1 ICB, we performed scRNA-Seq analysis of the CD45^^+^^ TICs in melanoma-bearing *Trem1^+/+^* mice receiving the various treatments. We analyzed approximately 8,249 CD45^^+^^ cells from the treatment groups with t-SNE analysis, identifying 10 distinct clusters of tumor-infiltrating immune cells (Figure 6A). We used the top 195 differentially upregulated genes in each cluster paired with a comparable data set from ImmGen for annotating the cell populations. Both VJDT as well as combinational therapy induced marked remodeling of the TME (Figure 6B). This included severely restricting both infiltrating macrophages (Cluster 1) expressing *Ccr2* and *Marco* (Figure 6C) as well as MDSCs showing Arg2 and Nos2 expression (Figure 6D). Among the lymphoid clusters, combinational treatment led to a marked increase in activated CD8^^+^^ T cells (Cluster 5) expressing *Gzmk* and *Gzmb* (Figure 6E), while attenuating exhausted CD4^^+^^ and CD8*^*^+^*^* T cells (Cluster 6 and 7) characterized by *Tigit, Lag3,* and *Ctla4* expression (Figure 6F). Additionally, VJDT monotherapy induced expansion of a unique group of unconventional αβ T (UTC__αβ__) cells (Cluster 3) expressing *Tmem176a-b*, *Rorc,* and *Il17a* (Figure 7A). To ensure the accurate annotation of these unconventional cells, we selected the top 50 genes differentially expressed by UTC__αβ__ cells (53). We screened this gene list across our scRNA-Seq expression data and identified Cluster 3 as most likely to be comprised of UTC__αβ__ (Supplemental Figure 4A). Flow cytometry assessment of the TME demonstrated that these UTC__αβ__ cells were slightly increased during monotherapy with anti-PD-1, and markedly increased during TREM1 inhibition (Figure 7B and Supplemental Figure 3E). In an effort to better characterize this population, we reclustered these tumor-infiltrating UCT__αβ__ cells, which were a highly heterogeneous population composed of distinct cell clusters showing molecular signatures for mucosal-associated invariant T (MAIT) cells, invariant natural killer T (iNKT) cells and αβ double negative T (DNT__αβ__) cells. Interestingly, VJDT monotherapy induced expansion of an iNKT cluster classified by the expression of *Cxcl10, Icos, Traj18,* and *Klra5,* while combinational treatment, which yielded the lowest tumor volumes, showed an increased presence of both MAIT cells and *Rorc*-expressing DNT__αβ__ cells (Supplemental Figure 4B). Moreover, the UTC__αβ__ population represented the dominant cluster within the TME, expressing proliferation markers such as *Mki67* and *Tuba1b* (Figure 7C), suggesting that this population preferentially expands in a TREM1-deficient TME, and that, via their plasticity and diversity, they can be potent drivers of antitumor immunity (53, 54). Simultaneously, scRNA-Seq data revealed an increased frequency of neutrophils (Cluster 9) expressing *Mmp8*, *S100a8,* and *S100a9* (35) during VJDT monotherapy and combination treatment (Figure 6B and Figure 7D). These neutrophils are selectively expanded in combinational treatment and could be a vital component of the potent antitumorigenic response of this therapeutic model. Further, increased neutrophil infiltration in the TREM1-deficient TME could be an integral driver for UTC__αβ__ cell recruitment and function synergistically to mediate a strong antitumorigenic immune profile (53, 54).

Overall, the heatmap of the normalized expression of selected genes in each group show that VJDT and combinational treatment inhibit genes essential for macrophage recruitment, *e.g.,* *Ccr2, Cx3cr1, and Ccl2,* as well as *Arg2* and *Nos2*, which are integral for MDSC immunosuppression. Additionally, combinational treatment enhanced overall T cell immunity while abrogating its exhaustion (Figure 7D). Further, anti-PD-1 monotherapy was the only treatment modality that showed marked upregulation in *Ido1*, a predictor for resistance to PD-1-based ICB therapy (55) (Figure 7D). In summary, data from our flow cytometry and scRNA-Seq analyses demonstrate that TREM1 inhibition alters the tumor immune landscape to limit MDSC frequency while boosting effector CD8^^+^^ T cell immunity to effectively augment PD-1 ICB therapy. Together, these results may form the basis of a promising approach for cancer therapeutics.

### Silencing TREM1 in human tumor cells attenuates tumor growth in xenograft models and decreases cell proliferation and cell motility.

**Figure 8:**
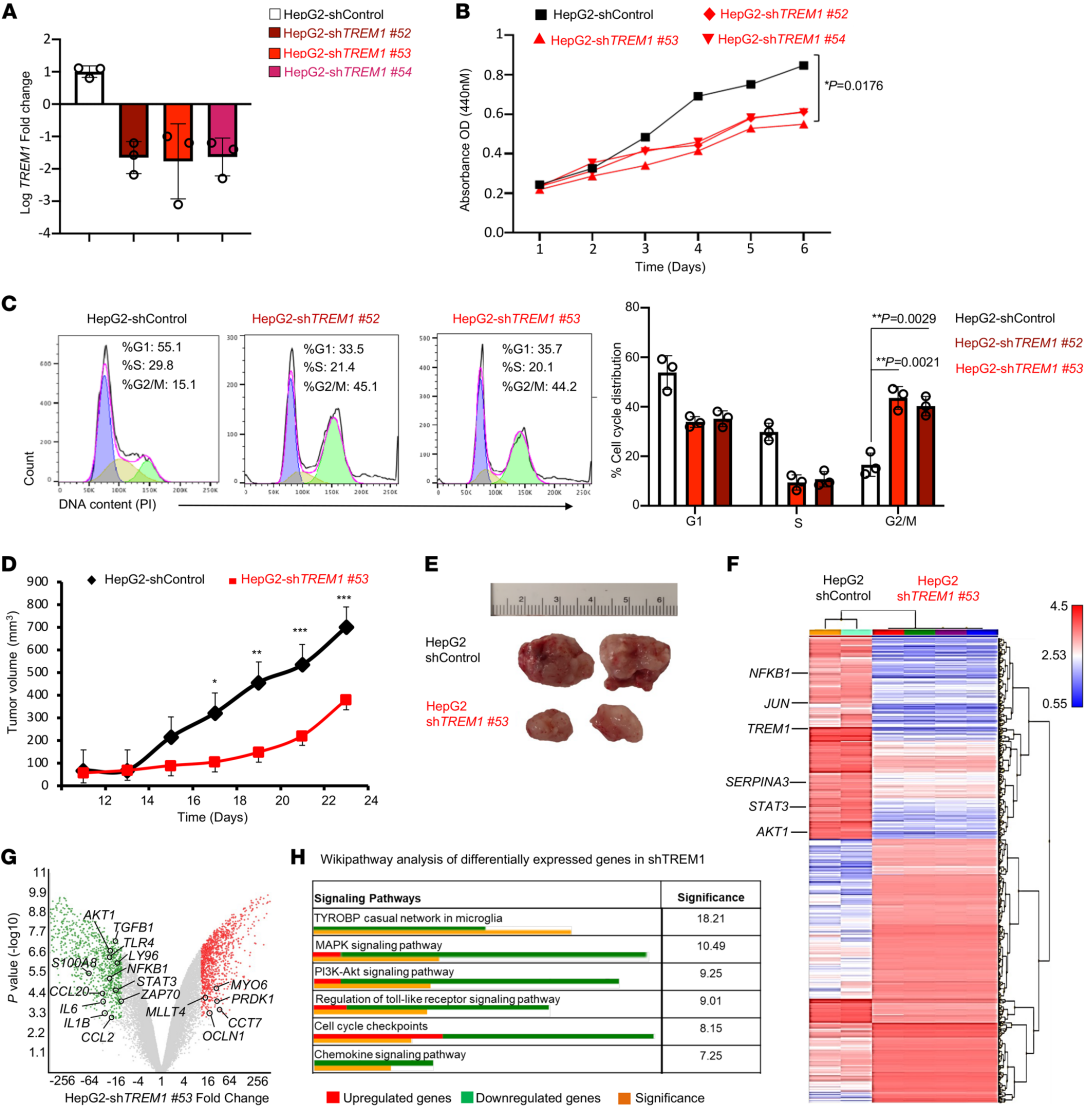
TREM1 silencing in HepG2 cells inhibits cell proliferation, migration, and tumor growth in xenograft model. (**A**) RT-qPCR confirmation of *TREM1* knockdown in HepG2 cells following transfection with sh*TREM1* clones nos. 52, 53, and 54 in comparison to shControl-scrambled vector. Data represent 3 independent experiments performed in triplicate (*n* = 3/group, mean ± SD). (**B**) Line graph shows WST-1 assay to assess cell proliferation in *TREM1* knockdown HepG2 clones (sh*TREM1* nos. 52, 53, and 54) and its shControl clone for 6 days (*n* = 3/group, mean ± SD). (**C**) Flow cytometry histogram plots depict cell cycle progression of *TREM1* knockdown HepG2 clones (sh*TREM1* nos. 52 and 53) and shControl over 24 hours. Representative plot from 3 independent experiments performed in triplicate (mean ± SD shown). (**D**) Tumor growth curves for *TREM1* knockdown sh*TREM1* clone no. 53 in NSG mice and its shControl described as overall tumor volume measured every alternate day (*n* = 8 mice/group, mean ± SEM). (**E**) Representative microscopic images of tumors from the indicated groups at day 23. (**F**) Transcriptomic analysis by Clariom S Microarray used to plot heatmap depicting hierarchical clustering of differentially expressed genes between sh*TREM1* no. 53 knockdown tumors (*n* = 4) and the shControl HepG2 tumors (*n* = 2). (**G**) Volcano plots depict differentially expressed genes of the sh*TREM1* no. 53 knockdown tumors compared with shControl HepG2 tumors. Red dots represent upregulated genes with fold change greater than 10 and *P* < 0.001, green dots show downregulated genes. (**H**) Wikipathway analysis depicts significantly affected pathways in *TREM1* knockdown HepG2 clone in comparison to control. *P < 0.05; **P < 0.01; ***P < 0.001 by 2-way ANOVA with Tukey’s correction *t* test for comparing cell proliferation (**B**), by 2-tailed Student’s *t* test for comparison between 2 groups (**C**), 2-way ANOVA for multiple comparison of longitudinal tumor growth between various groups (**D** [tumor growth]), or using 2-sided Fisher’s exact *t* test in pathway analysis (**H**).

TREM1 expression is closely associated with tumorigenesis in selected human tumors (7, 19). Although the intrinsic role of TREM1 in cancer cells remains unclear, we have observed TREM1 expression in several human cancer cell lines, such as HepG2, Huh7, U87, and U251 (Supplemental Figure 5A). To better characterize the role of TREM1 expressed intrinsically in cancer cells, we established several stable HepG2 clones with confirmed TREM1 knockdown through RT-qPCR (Figure 8A). Interestingly, cell proliferation was attenuated in all the HepG2 clones with *TREM1* knockdown compared with the clones treated with the scrambled controls (Figure 8B). Cell cycle analysis of 2 of these knockdown HepG2 clones (no. 52 and no. 53) revealed significant G2/M cell cycle arrest (Figure 8C); further, inhibition of TREM1 by VJDT treatment at 50 μM similarly induced G2/M arrest, while 10 μM treatment induced S phase arrest in HepG2 cells (Supplemental Figure 5B). The observed G2/M arrest in TREM1-deficient cells is consistent with the described role of TREM1 in modulating the energy metabolism required for the G2-to-M transition under stress conditions (56). Furthermore, pharmacological inhibition of TREM1 by VJDT treatment significantly attenuated cell proliferation and migration in HepG2 and B16F10 cells (Supplemental Figure 5, C–F). Interestingly, VJDT inhibited the growth of B16F10 tumors implanted in *Trem1^–/–^* mice; however, their effect on TICs was minimal (Supplemental Figure 6, A and B). These results suggested that pharmacological inhibition of TREM1 can directly restrain tumor growth independent of its role in modulating the TME. To further characterize the tumor-promoting role of TREM1 in vivo, we performed xenograft studies using immunodeficient NSG mice. We validated that knockdown of *TREM1* on HepG2 cells significantly restricted tumor growth compared with the control group (Figure 8, D and E). The transcriptomic expression profiles of sh*TREM1* and shControl HepG2 cells were compared by microarray analysis. Knockdown of *TREM1* led to downregulation of key genes involved in cell proliferation, such as *JUN*, *STAT3*, *NFKB1*,and *AKT1* (Figure 8, F and G). Other genes inhibited by *TREM1* knockdown included chemokines such as *CCL2* and *CCL20,* which are involved in macrophage recruitment (57, 58), and cytokines *IL1B*, *IL2,* and *IL6*, which play a pivotal role in inflammatory responses (59–61). Wikipathway analysis of sh*TREM1* HepG2 tumors revealed significant downregulation of the PI3K-Akt and MAPK signaling pathways (Figure 8H), which are critical for oncogenesis (62). Additionally, *TLR4* and *LY96*, which are genes in the TLR signaling pathway that promote cell survival and proliferation in hepatocellular carcinoma (63, 64), were significantly downregulated in sh*TREM1* HepG2 tumors (Figure 8, G and H).

### High TREM1 expression is associated with poor prognosis in human cancers, and TREM1 inhibition reduces tumor burden in melanoma patient–derived xenografts.

**Figure 9:**
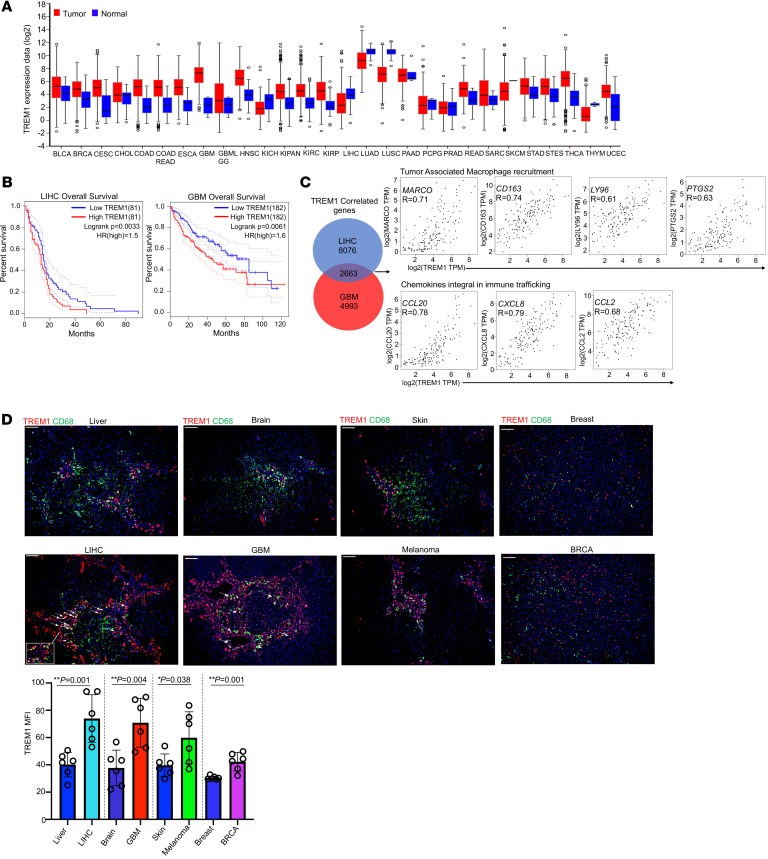
TREM1 expression is associated with poor prognosis in selected human tumors. (**A**) *TREM1* mRNA expression between 28 human neoplastic and corresponding nonneoplastic tissues. (**B**) Kaplan-Meier survival curves describe correlation between *TREM1* expression and overall patient survival in LIHC (*n* = 162) and GBM (*n* = 364) cohorts. (**C**) Venn diagram showing *TREM1*-correlating genes in LIHC and GBM cohorts. Correlation data for *TREM1* expression (X axis) and the indicated genes (Y axis) for LIHC cohort. (**D**) Fluorescent multiplex IHC images of human samples from people in the control group and people diagnosed with primary carcinomas in liver, brain, skin, and breast. Original magnification, ×10; scale bar: 100μm. TREM1 (red), DAPI (blue), and CD68 (green). Quantification of overall TREM1 expression as MFI in different zones of tumor and control tissues (*n* = 6 zones per tissue section). *P < 0.05; **P < 0.01 by 2-tailed Student’s *t* test for comparison between 2 groups.

We explored the Cancer Genome Atlas (TCGA) database to assess *TREM1* expression in human tumors and normal tissues. As expected, *TREM1* transcript levels were elevated in almost all human tumor cohorts compared with their corresponding normal tissues (Figure 9A). We next examined the association between *TREM1* expression and overall survival by utilizing the Cox regression model on the various tumor types. With a 50% quantile as cutoff for *TREM1* expression, only the LIHC and GBM cohorts exhibited significant associations between high *TREM1* expression and worse overall survival (Figure 9B). To better understand the impact of *TREM1* in these 2 carcinomas, we identified genes that correlated with *TREM1* expression in both tumor types. High positive correlations were identified between *TREM1* expression and several chemokines integral to immune trafficking (65, 66) (*CCL20*, R=0.78; *CXCL8*, R=0.79; and *CCL2*, R=0.68), as well as genes involved in myeloid cell recruitment within the TME (67–70) (*MARCO*, R=0.78*; CD163*, R=0.71; *LY96*, R=0.61; and *PTGSR*, R= 0.63; Figure 9C). To further validate findings from the TCGA database, we analyzed TREM1 protein expression in selected human normal and tumor tissue sections by immunofluorescence staining. We detected significantly elevated TREM1 protein within the TME of LIHC, GBM, breast cancer, and melanoma (Figure 9D). TREM1 expression in LIHC was concentrated around the macrophage-specific CD68 marker, implying the presence of infiltrating TREM1^^+^^ macrophages in these tumors. Other tumors, such as GBM, exhibited high levels of TREM1 expression independent from their CD68^^+^^ microglial cells (Figure 9D), indicating that these neoplasms were intrinsically TREM1 positive. Together, our results highlight that TREM1 expression is associated with various human carcinomas and plays a critical role in modulating tumor immune infiltrates, making it a candidate for therapeutic intervention.

**Figure 10:**
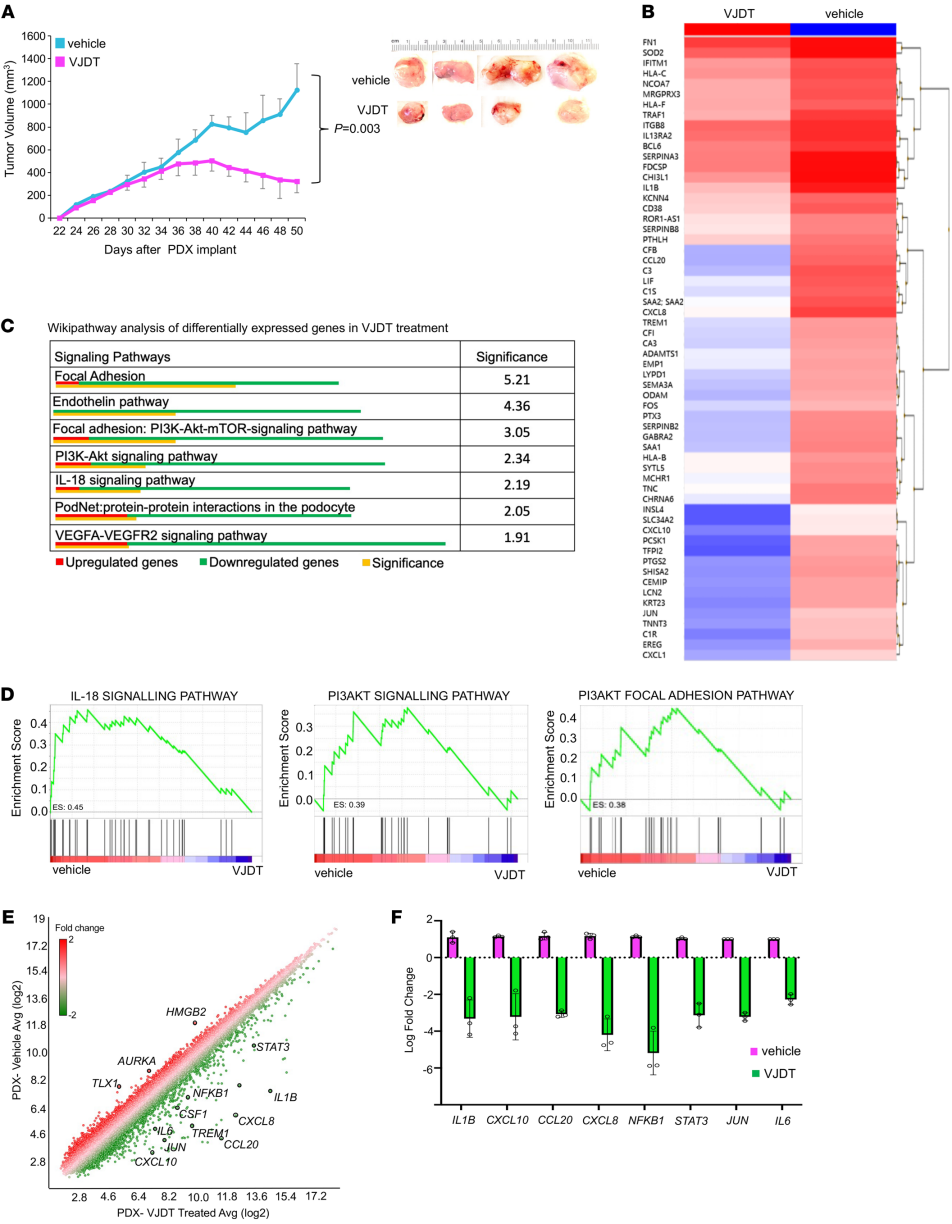
TREM1 inhibition by VJDT treatment restrains tumor growth in PDX models. (**A**) Tumor growth curve of PDX melanoma xenograft in NSG mice receiving vehicle (DMSO) or VJDT (20 mg/kg) treatment every alternate day from 30 to 48 days (*n* = 4 mice/group, mean ± SEM). (**B**) Heatmap depicts hierarchical clustering of differentially expressed genes in PDX tumors between VJDT-treated versus vehicle. (**C**) Wikipathway analysis identify signaling pathways in PDX tumors significantly affected by VJDT treatment. (**D**) GSEA analysis showing downregulated signaling pathways during VJDT treatment. Enrichment score (ES) are shown. (**E**) Scatter plot depicts expression profile of specific genes of interest in VJDT-treated versus vehicle control. (**F**) Custom RT-qPCR confirmation of expression profile for key genes altered by VJDT treatment. Data from 3 independent experiments performed in triplicate (mean ± SD shown). *P* value calculated by 2-way ANOVA with Tukey’s correction *t* test for multiple comparison of longitudinal tumor growth between various groups (**A** [tumor growth]) or using 2-sided Fisher’s exact *t* test in pathway analysis (**C**).

We further evaluated the in vivo efficacy of the small molecule TREM1 inhibitor VJDT against a human skin cutaneous melanoma PDX model. NSG mice bearing PDX tumors were randomized to treatment with vehicle or VJDT (20 mg/kg). Compared with the control group, the treatment with VJDT significantly suppressed the growth of melanoma PDX tumors (Figure 10A). To identify potential targets of VJDT, we performed microarray gene expression profiling on melanoma PDX tumors treated with VJDT compared with untreated (Figure 10B). We next performed pathway analysis to identify the critical signaling networks most affected by VJDT treatment. Remarkably, many cellular proliferation and migration signaling pathways, such as the PI3K-Akt and the PI3K-Akt-mTOR focal adhesion pathways, were inhibited by VJDT (Figure 10C), suggesting that TREM1 contributes to tumorigenesis. In addition, GSEA showed selective enrichment of key genes involved in the IL-18, PI3K-Akt, and PI3K-Akt focal adhesion pathways in the vehicle group, which was abrogated by VJDT treatment (Figure 10D). Marked downregulation of expression of *TREM1* and known TREM1 target genes (*NFKB1*, *CCL20, IL6,* and *CXCL8*) was determined in melanoma PDX models treated with VJDT (Figure 10E). Further, VJDT treatment strongly downregulated genes associated with cell proliferation (*STAT3, NFKB1,* and *JUN*) and those associated with immune cell infiltration (*CCL20, CXCL8, CXCL10,* and *IL1B*) (71–73) (Figure 10E). The regulation of expression of selected genes was validated by quantitative real-time PCR analyses (Figure 10F). Together, these results indicate a global suppression of genes essential for human tumorigenesis during pharmacological inhibition of TREM1 and support the potential therapeutic usefulness of VJDT.

## Discussion

TREM1 has increasingly been recognized as a central player in various types of malignancies in addition to its well-established role in inflammatory disorders (11, 20, 74, 75). Here, we show that TREM1 modulates TME and MDSC functions, acts as a tumor oncoprotein, and is a potential cancer therapeutic target. Genetic silencing of TREM1 or its pharmacological inhibition using the newly developed inhibitor VJDT restrains tumor growth, and, in combination with anti-PD-1 treatment, results in complete tumor regression. TREM1 silencing substantially remodeled the TME of B16F10 tumors by targeting myeloid cells, specifically attenuating MDSC accumulation and immunosuppressive capacity. scRNA-Seq analysis revealed that TREM1 inhibition led to decreased frequency of CCR2-expressing tumor-infiltrating myeloid cells as well as downregulation of the CCR2/CCL2 axis and CX3CR1 and CXCL2 chemokines, which are key players in the tumor-homing pathways responsible for MDSC accumulation in the TME (76, 77). These observations support and extend our prior discoveries that TREM1 deficiency attenuates development of liver fibrosis/injury by selectively targeting recruitment of proinflammatory monocytes (6). Within the TME, MDSCs suppress T cell activity by several mechanisms, including the expression of arginase and iNOS for production of nitric oxide, the generation of ROS, and depletion of the TME of nutrients critical for T cell function. Of particular interest is ROS generation by the NADPH oxidase isoform NOX-2. These ROS can prevent a T cell receptor (TCR)/MHC-peptide interaction by catalyzing nitration of the TCR/CD8 molecule (47, 78). GSEA of scRNA-Seq data revealed that TREM1 silencing in MDSCs led to a decrease in gene signatures involved in ROS production and oxidative phosphorylation pathways. Furthermore, the diminished functions of MDSCs from *Trem1*^^–/–^^ mice were paralleled by a global attenuation in markers of immunosuppressive function, such as *Arg1, Nos2,* and *Nos1* (79, 80). Mki67-expressing cycling macrophages were depleted during TREM1 deficiency, indicating a TREM1 prosurvival role, which was previously established in neutrophils and monocytes (81, 82). Thus, targeting TREM1 may provide a unique therapeutic approach by decreasing the accumulation, functions, and survival of MDSCs in the TME while simultaneously increasing the efficiency of current chemotherapy, radiotherapy, and immunotherapy protocols.

Within the lymphoid compartment of the TME, TREM1 genetic or pharmacologic inhibition led to an increase in CD8^^+^^ T cell activation while expanding the subpopulation of CD8^^+^^ T cells expressing high levels of PD-1. The mechanisms by which TREM1 deficiency affects these TILs remain unclear. It is likely that the reduction of MDSC populations and their immunosuppressive function observed in the *Trem1^–/–^* TME is one of the potential mechanisms for enhancing CD8^^+^^ T cell expansion and the expression of PD-1 on those cells, making them more susceptible to anti-PD-1 treatment. Previous studies demonstrated that reprogramming the immunosuppressive capacity of MDSCs or inhibiting its biogenesis (83) enhances the response to anti-PD-1 therapy in melanoma (84) and colorectal cancers (85, 86). In the current study, combining TREM1 inhibition and anti-PD-1 ICB induced complete tumor regression. Additionally, our scRNA-Seq data demonstrate a novel recruitment of antitumorigenic UTC__αβ__ cells in the TME during pharmacological inhibition of TREM1. Simultaneous expansion of tumor-infiltrating neutrophils in the same treatment groups suggest a previously unknown mechanism by which TREM1 deficiency drives neutrophil-dependent expansion of UTC__αβ__ antitumorigenic immunity and warrants further research. It is possible that genetic deletion of *Trem1* — and especially pharmacological inhibition of TREM1 — can induce differentiation of pathologically activated PMN-MDSCs toward terminally differentiated neutrophils with the ability to expand/polarize UTCs and display antitumor potential, as we demonstrated in our presented studies. ICB therapies such as anti-PD-1 show unprecedented durable response rates in clinical conditions. However, their efficacy is limited in patients due to the development of an immunosuppressive TME. The results of our study (a) demonstrate a synergistic effect where pharmacological inhibition of TREM1 remodels the TME, sensitizing it to anti-PD-1 treatment, and (b) provide a viable therapeutic strategy to overcome resistance in immunotherapy.

In addition to a role of TREM1 expression on immune cells, there has been growing interest on the role of TREM1 as a driver of oncogenesis in cancer cells (20, 74, 87, 88). Analysis of TCGA database revealed elevated TREM1 expression in numerous human carcinomas compared with normal tissue cells. However, TREM1 expression negatively correlated with patient survival only in LIHC and GBM cohorts. This implies that the role of TREM1 in patient survival might be context-specific and tumor-dependent. Correlation analysis using both LIHC and GBM cohorts revealed that *TREM1* expression was associated with macrophage recruitment and immune-trafficking gene signatures. Our study demonstrates that targeting TREM1 expression in human HepG2 cells inhibits cell proliferation by interfering with signaling pathways such as PI3K-Akt and MAPK. Further, by utilizing the melanoma PDXs, we demonstrated that VJDT treatment pharmacologically inhibits TREM1 and curbs tumor growth. Essential signaling networks for tumor maintenance and progression, such as MAPK, PI3K, and the PI3K-Akt adhesion pathways, were globally inhibited in VJDT-treated PDX tumors. Most interestingly, TREM1 inhibition led to substantial attenuation of the IL-18 signaling pathway, which is a proinflammatory cytokine network associated with immune cell infiltration in various tumors. These data indicate the possibility of a novel TREM1-dependent, IL-18 signaling network that may suppress an antitumorigenic immune response.

In this study, we analyzed the impact of TREM1 in tumor progression utilizing a combination of *Trem1^–/–^* mice, silencing of TREM1 in human cancer cells, and pharmacological inhibition of TREM1 via the small molecule inhibitor VJDT in several mouse tumors and a melanoma PDX model. Our work highlights the ability of TREM1 inhibition in diminishing tumor progression and augmenting the efficacy of PD-1 immune checkpoint blockade. We provide supporting observations to the previously unrecognized role of TREM1 in MDSC accumulation and function within the TME. Our observations highlight a dual role of TREM1 as an oncogene in cancer cells and an immunosuppressor gene in TME myeloid cells, suggesting that TREM1 has the potential to be a novel target for cancer treatment. Further studies on mechanisms controlling oxidative stress pathways within MDSCs by TREM1 could lead to future treatment strategies to selectively deplete the immunosuppressive TME and enhance the response of ICB therapies.

## Methods

### Mice.

*Trem1-*KO mice on a C57BL/6J genetic background were generated as described previously (6). Breeding of *Trem1* heterozygous parents (*Trem1^+/–^*) yielded *Trem1^–/–^* and *Trem1^+/+^* offspring. PCR genotyping was performed based on the product sizes 300 and 506 bp for the *Trem1^+/+^* and mutant alleles, respectively. The ratio of *Trem1*^^–/–^^, *Trem1*^^+/–^^, and *Trem1^+/+^* offspring did not deviate from the expected 1:2:1 ratio. All animals had normal weight and appearance. *Trem1* expression was analyzed by PCR amplification of a 152-bp product using forward (5′-CGCCTGGTGGTGACCAAGGG-3′) and reverse (5′-ACAACCGCAGTGGGCTTGGG-3′) primers. *Trem1^+/+^* littermates were used as control for in vivo tumorigenesis studies. NOD *scid* γ (NOD.Cg-*Prkdc^^scid^^Il2rg^^tm1Wjl^^/*SzJ, JAX 005557) mice, 6–8 weeks of age, were purchased from the Jackson Laboratory. Age- and sex-matched animals were included in all experiments.

### Tumor models and treatments.

8-week-old *Trem1^+/+^* or *Trem1^–/–^* mice were s.c. injected with syngeneic 1 × 10^^5^^ B16F10 murine melanoma cells or 1 × 10^^6^^ MCA205 fibrosarcoma cells into their right flanks. Tumor growth was measured alternate days using a digital caliper; tumor volumes were calculated using the formula V=L × W^^2^^ × (π/6), where L and W denote length and width of the tumor. For pharmacological inhibition of TREM1, 20 mg/kg VJDT or vehicle (DMSO) were administered intraperitoneally in *Trem1^+/+^* mice on day 8 after tumor cell injection and continued alternate days until day 20. For specific groups, anti-PD-1 treatment was performed with 200 μg anti-PD-1 antibody (BioXCell, BE0273, clone: 29F.1A12) or the corresponding IgG2a isotype control (BioXCell, BE0089, clone: 2A3) injected i.p. alternate days from days 8 to 20 of tumor growth. Mice were euthanized on day 22 after tumor cell injection. In cell line–derived xenograft studies, 1 × 10^^6^^ *TREM1* knockdown clones of HepG2 cells or vector control cells were s.c. injected into the right flank of 6-to-8 week-old immunodeficient NSG mice. Tumor volume was measured alternate days as described previously; mice were euthanized on day 24 of tumor growth. For PDX studies, patient-derived melanoma xenograft tumor models were purchased from JAX Mice, Clinical and Research Services (Jackson Laboratory, TM00943). Tumor growth was measured alternate days and volume calculated as described previously. Pharmacological inhibition of TREM1 was performed by VJDT treatment (20 mg/kg) or vehicle (DMSO) by i.p. injections from days 30 to 48 of tumor growth. Mice were euthanized on day 50 of tumor growth.

### Flow cytometry analysis of MDSCs.

Freshly harvested tumors from *Trem1^+/+^* or *Trem1^–/–^* mice were processed into a single-cell suspension using gentleMACS Octo Dissociator with Heaters (Miltenyi Biotech) in combination with the tumor dissociation kit (Miltenyi Biotech). Cells were stained with fluorochrome–conjugated antibodies according to the manufacturer’s instructions. For surface staining, cells were prepared and suspended in PBS and incubated with following antibodies (all from BioLegend) at 4°C for 45 minutes in dark: TruStain FcX (clone: 93, 101319, 1:50 dilution), anti-F4/80-APC (clone: BM8, 123116, 1:100 dilution), anti-F4/80-FITC (clone: BM8, 123108, 1:100 dilution), anti-CD11b-APC (clone: M1/70, 101212, 1:100 dilution), anti-CD11b-PE (clone: M1/70, 101208, 1:200 dilution), anti-CD11b-APC/Cy7 (clone: M1/70, 101226, 1:100 dilution), anti-Gr1-APC/Cy7 (clone: RB6-8C5, 108423, 1:100 dilution), anti-Ly6C-PE (clone HK1.4, 128007, 1:200 dilution), anti-Ly6C-APC/Cy7 (clone: HK1.4, 128026, 1:100 dilution), anti-Ly6G-PE (clone: 1A8, 127608, 1:200 dilution), and anti-Ly6G-APC/Cy7 (clone: 1A8, 127624, 1:100 dilution). Cells were acquired on the Attune NxT Acoustic Focusing flow cytometry platform (Thermo Fisher Scientific) and data were analyzed on FlowJo v10.0.

### Flow cytometry analysis of T cells.

Tumors were processed as described in the above section for MDSC analysis and were incubated with the following antibodies (all from BioLegend) at 4°C for 45 minutes in the dark: TruStain FcX (clone: 93, 101319, 1:50 dilution), anti-CD4-APC (clone: RM4-5, 100516, 1:100 dilution), anti-CD3-FITC (clone: 17A2, 100204, 1:100 dilution), anti-CD8-PerCP (clone: 53-5.8, 140417, 1:200 dilution), and anti-CD25-APC/Cy7 (clone: 3C7, 101918, 1:100 dilution). Following staining, cells were fixed and permeabilized using the Cyto-Fast Fix/Perm Buffer (BioLegend, 426803) according to manufacturer’s protocol and stained intracellularly with following antibodies: anti-TNFα-PE (eBioscience, clone: MP6-XT22, 12-7321-81, 1:200 dilution), anti-TGF-β1-PE (BioLegend, clone: TW7-16B4, 141403, 1:200 dilution), anti-IFN-γ-PE (BioLegend, clone: XMG1.2, 113603, 1:100 dilution), anti-IL-2-PE (eBioscience, clone: JES6-5H4, 12-7021-81, 1:200 dilution), anti-IL-10-PE (BioLegend, clone: JES5-16E, 3505007, 1:200 dilution), anti-IL-17-PE (BioLegend, clone: TC11-18H10.1, 506903, 1:200 dilution), anti-IL-1b-PE (eBioscience, clone: NJTEN3, 12-7114-80, 1:200 dilution), and anti-Granzyme B-FITC (BioLegend, clone: GB11, 506903, 1:200 dilution). All samples were acquired on the Attune NxT Acoustic Focusing flow cytometry platform (Thermo Fisher Scientific). Further analysis was performed on FlowJo v10.0. Forward versus side scatter (FSC versus SSC) gating were used to exclude dead cells.

### scRNA-Seq sample preparation.

Freshly harvested tumors from *Trem1^+/+^* and *Trem1^–/–^* mice were pooled separately into 2 groups to maximize isolation of tumor infiltrating myeloid cells. Tumors were dissociated using gentleMACS Octo Dissociator with Heaters (Miltenyi Biotech). Single-cell suspensions were purified by incubation with CD45 (Miltenyi Biotec, 130-110-618) and CD11b Microbeads (Miltenyi Biotec, 130-049-601) per manufacturer’s instructions to enrich tumor-infiltrating myeloid cells. Quality and quantity of cells were assessed by trypan blue staining; dead cells were removed with dead cell removal kit (STEMCELL) per the manufacturer’s instructions. To capture 5,000 targeted cells, 7,000 to 8,000 live myeloid cells were loaded onto the Chromium Controller (10× Genomics), and scRNA-Seq libraries were generated using Chromium Next GEM Single Cell 3′ Reagent kit v3.1 (10× Genomics) according to the manufacturer’s protocol. The libraries were sequenced on NextSeq 500 (Illumina) using Mid Output v2.5 (150 cycles) kit (Illumina) with 28 bp (Read 1), 8 bp (Indexing Run), and 91 bp (RNA Read 2) to collect approximately 23 K mean reads per cell at the range of 1,294,379 median genes per cell. Reads from the raw fastq files were mapped to the mm10 mouse genome reference by STAR aligner linked with Cell Ranger v5.0.1 pipeline to output clusters representing the cell populations. Upregulated genes were annotated as the cell surface markers in each population. The output was imported into Loupe Browser v6.0 (10× Genomics) for visualization and further analysis.

### scRNA-Seq data processing.

Loupe Browser v6.0 (10× Genomics) and Partek Flow (Partek) were utilized for data processing and analysis of the scRNA-Seq results. Initially, cells with high levels of mitochondrial genome transcript reads were filtered out. Additionally, all cells were confirmed to actively express *Ptprc* (CD45). Principal component analysis (PCA) and dimensional reduction were performed using Cluster function based on the top globally distinguishing genes among the cell populations. Classification and cell annotation were performed by comparing the expression profile of each cluster with a correlating data set from the ImmGen Consortium and the expression of classical gene markers. Briefly, *Marco* and *Ly6c2* were used for broad identification of macrophage/monocytic populations consisting of infiltrating monocytes expressing *Ccr2* and *Cx3cr1* (28, 33)*,* TAMs positive for *Cd68* and *Cd163* (17, 89), tumor-infiltrating macrophages with high levels of *Irf8* expression (31, 42), and cycling macrophages positive for *Mki67* (32). High expression of *Ly6g2*, *S100a8,* and *MMP8* were used to classify neutrophilic populations consisting of *MMP8* neutrophils and infiltrating neutrophils expressing *Cd163, Ly6g2,* and *Il6* (35). MDSCs defined as *Arg2, Nos2*, *Il1b, Stat3,* and *Cd84*–expressing cells were diffusely represented in various clusters. The top locally distinguishing gene set per cluster was extracted and utilized for GSEA analysis, as described previously. In experiments where the entirety of CD45^^+^^ TICs were utilized for scRNA-Seq, the lymphoid clusters were classified based on expression of activation markers such as *Gzmk* and *Gzmb* (28) or exhaustion markers such as *Tigit, Lag3,* and *Ctla4* (28, 30). UTC__αβ__ clusters were separately classified based on contiguous expression of 50 gene markers in specific clusters (53).

### T cell suppression assay.

Tumor-infiltrating MDSCs were isolated from freshly harvested B16F10 tumors of *Trem1^+/+^* or *Trem1^–/–^* mice using the MDSC isolation kit (Miltenyi Biotec) per the manufacturer’s instructions. The purity of MDSCs was over 80%–90% as verified by flow cytometry. CD3^^+^^ T cells were harvested from spleens of *Trem1^+/+^* mice and enriched by negative selection via Pan T cell isolation kit (Miltenyi Biotec) per the manufacturer’s instructions. The purified CD3^^+^^ T cells were stained with CFSE (Thermo Fisher Scientific, C34570, 2 μM) and maintained in complete medium consisting of RPMI (STEMCELL Technologies), 10% heat-inactivated FBS (Hyclone),100 U/mL penicillin, and 100 μg/mL streptomycin (Corning). For proliferation, CD3^^+^^ T cells were primed using anti-CD3/CD28 beads (Gibco, 11-452-D) for 72 hours according to manufacturer’s recommendation and cocultured with purified MDSCs at a 2:1 ratio. T cell proliferation was subsequently measured by acquiring the extent of CFSE dilution in T cells on an Attune NxT Acoustic Focusing flow cytometry platform (Thermo Fisher Scientific) and analyzed using FlowJo v10.0.

### ROS detection.

MDSCs were isolated from *Trem1^+/+^* or *Trem1^–/–^* tumor-bearing mice as described earlier. ROS were detected using the ROS assay Kit (Invitrogen) per the manufacturer’s instructions. For induced activation experiments, MDSCs were cocultured with 30 ng/mL PMA (Thermo Fisher Scientific, 50-058-20001). ROS formation was acquired on an Attune NxT Acoustic Focusing flow cytometry platform (Thermo Fisher Scientific) and analyzed using FlowJo v10.0.

### Lentiviral TREM1 knockdown in HepG2 cells.

TREM1 knockdown was performed using 5 TREM1 shRNA–containing lentiviral vectors purchased as the Human pLKO.1 Lentiviral Human TREM1 shRNA target gene set (Horizon Discovery, RHS4533-EG54210). The target sequences were: sequence 1 AAGGTTGATTTCAGAGTCAGG, sequence 2 ATCTTCCACTTGAAGGTTGAC, sequence 3 TAGGGTACAAATGACCTCAGC, sequence 4 ATTATCTGCCAAGCTTTCTGG, and noncoding control sequence 5 AATGACAATGTTGAACACCGG. The 5 glycerol stocks of *Escherichia coli,* each containing the pLKO.1 vector with individual shRNA constructs targeting the human *TREM1* gene or nontargeted scrambled control, were propagated in LB agar plates and grown in terrific broth (Thermo Fisher Scientific). Plasmid DNA from bacterial growth was isolated using QIAprep Spin Miniprep kit (Qiagen). Lentiviral preparation and packaging were performed using the Trans-Lentiviral shRNA packaging kit (Perkin Elmer) per the manufacturer’s instructions. Finally, HepG2 cells were transduced with pseudo-lentiviral particles, and transfected HepG2 cells were enriched by growth in puromycin-containing (Sigma-Aldrich, 5 μg/mL) selection medium.

### Signaling pathway analysis and GSEA.

CEL output data files from microarray gene expression profiling were further analyzed. Signaling pathway estimation and scatter plot depiction were performed using Transcriptome Analysis Console v4.0 (Applied Biosystems). The significant gene list of each sample group was used for GSEA analysis utilizing the curated gene sets of the Molecular Signature Database v4.0 (MsigDB) provided by Broad Institute (http://www.broad.mit.edu/gsea) according to GSEA user guide (http://www.broadinstitute.org/gsea/doc/GSEAUserGuideFrame.html). The FDR for GSEA is the estimated probability that a given normalized enrichment score represents a false-positive finding, and an FDR under 0.25 is considered to be statistically significant for GSEA.

### TREM1 expression profiling in TCGA database.

TREM1 expression profiling was performed across both the TCGA database and Genotype Tissue Expression (GTEx) projects containing the mRNA expression data in various types and stages of cancer. We used Gene Expression Profile Interactive Analysis (GEPIA) (http://gepia.cancer-pku.cn/), a web server for analyzing RNA-Seq expression data of TREM1 across different cancer tissues. Additionally, GEPIA was employed to conduct Kaplan-Meier survival analyses for TREM1 expression across all available tumors. Patient samples were classified into high- and low-expressing groups based on 50% quantile of *TREM1* expression. The expression correlation between TREM1 and relevant genes of interest was evaluated using the GEPIA database. An R value greater than 0.1 was selected as a positive association, and a *P* value under 0.05 was the criteria for statistical significance.

### Statistics.

Statistical analysis was performed using GraphPad Prism 9 (v.9.0.0). Data are presented as mean ± SD, if not otherwise stated. Graphs represent either group mean values ± SD (for in vitro experiments) or ± SEM (for in vivo experiments) or individual values. If data sets followed a normal distribution and comparisons were done between 2 experimental groups, then unpaired, 2-tailed Student’s *t* test was used. For in vitro studies, statistical comparisons were made with unpaired *t* tests when comparing 2 groups, and for in vivo studies, 2-way ANOVA was used for the multiple comparison of longitudinal tumor growth between various groups. *P* < 0.05 was considered statistically significant. Statistical analyses were implemented in consultation with the Biostatistics and Data Science Division of Augusta University.

### Study approval.

The IACUC of Augusta University approved the study protocol (2008-0051). Animals were housed in a climate-controlled specific pathogen-free environment within the Augusta University animal facilities. Experimental animals were provided with standard rodent food supplemented with grain and water ad libitum.

### Data availability.

The data generated or analyzed are included in this manuscript and its supplemental files. Single-cell sequencing data are deposited at EMBL-EBI ArrayExpress and are available under the accession numbers E-MTAB-13237, E-MTAB-13246, and E-MTAB-13300. The microarray data is available under accession numbers E-MTAB-13249 and E-MTAB-13257. Values for all data points in graphs are reported in the Supporting Data Values file. All other data relevant to the current study are available from corresponding authors upon reasonable request excluding confidential patent information.

## Author contributions

AA developed experimental protocols, designed, performed, and analyzed experiments, and wrote the manuscript. KM designed, performed, and analyzed experiments and wrote the manuscript. AM, AKD, and JRF performed and analyzed experiments. DDH, AC, XZ,and IL contributed to the experiments. GT designed and analyzed experiments, interpreted results, and wrote the manuscript. AH developed experimental protocols, directed the project, designed experiments, interpreted results, and wrote the manuscript.

## Supplementary Material

Supplemental data

Supporting data values

## Version 1 08/31/2023

In-Press Preview

## Version 2 11/01/2023

Electronic publication
