# Supplementary material for: Targeting TREM1 augments antitumor T cell immunity by inhibiting myeloid-derived suppressor cells and restraining anti–PD-1 resistance
Source: J Clin Invest. 2023 Nov 1;133(21):e167951. doi: 10.1172/JCI167951 (PMC10617775; doi:10.1172/JCI167951)
Supplement: Supplemental data [file jci-133-167951-s204.pdf]

Supplemental data

Targeting TREM1 augments antitumor T-cell immunity by inhibiting myeloid-derived suppressor cells and restraining anti-PD-1 resistance

Ashwin Ajith, Kenza Mamouni, Daniel D. Horuzsko, Abu Musa, Amiran K. Dzutsev, Jennifer R. Fang, Ahmed Chadli, Xingguo Zhu, Iryna Lebedyeva, Giorgio Trinchieri, Anatolij Horuzsko

Table of Contents

Supplemental Methods……………………………………………………………..….2

Supplemental References…………………………………………………………....11

Figure S1………………………………………………………………………….…..…12

Figure S2……………………………………………………………………………......14

Figure S3……………………………………………………………………………......17

Figure S4……………………………………………………………………………......18

Figure S5…………………………………………………………………..………….…19

Figure S6…………………………………………………………………..………….…21

Supplemental Methods

Design and development of TREM1 small molecule inhibitors: The development and design of VJDT, the novel small molecule inhibitor of TREM1, is detailed in the US patent application No.PCT/US21/51072 (patent pending). Receptor-based virtual screening of potential TREM1 inhibitors from the NCI Diversity Set was carried out using molecular docking analysis with AutoDock Vina v1.1.12 (Supplemental Figure S2A). A scoring system was used to discriminate compounds with the highest estimated affinity for the TREM1 binding site as described previously (1). The top 25 candidate compounds with higher binding mode to TREM1 were selected for additional screening using the TREM1 reporter cell lines stably transfected with TREM1 together with DAP12 and NFAT-driven GFP. In these reporter cells, TREM1 activation induces Ca^{2+}-driven signaling for NFAT nuclear translocation and subsequent NFAT-promoted synthesis of GFP, which in turn is detected by flow cytometry (Supplemental Figure S2B). The TREM1 reporter cells were activated by the anti-TREM1 agonistic antibody AF1278 (R&D Systems, AF1278) at a concentration of 4μg/ml for 24 hrs and served as positive control. For TREM1 blocking/inhibition studies, reporter cells were preincubated with each test compound at 50 μM for 6 hrs followed by stimulation with the anti-TREM1 agonistic antibody AF1278 for an additional 24 hrs. TREM1 activation in each blocking and agonist combination could be measured as a GFP signal acquired by an Attune NxT Acoustic Focusing flow cytometry platform (Supplemental Figure S2C). Based on the reporter cell line screening, we identified several candidate compounds exhibiting a TREM1 inhibitory effect and TREM1 agonist activity. From the

TREM1 antagonistic compounds, NCI118818 was selected as the most effective TREM1 inhibitor. Additionally, the blocking potency of TREM1 inhibitors (morin hydrate and NCI118818) were assessed via functional assay by measuring the reduction in cytokine production by TREM1-activated human neutrophils during inhibition treatment (Supplemental Figure S2D). For the functional assay, peripheral blood mononuclear cells (PBMCs) were isolated from healthy volunteers and stimulated with anti-TREM1 agonist antibody for 24 hours at 4 μg/ml or were pretreated with the indicated inhibitory compounds (at 50 μM) for 6 hrs followed by activation with anti-TREM1 agonist antibody for 24 hrs (Supplemental Figure S2D). Neutrophils were identified by staining with cell surface markers CD15 (BioLegend, clone: W6D3, 323005, 1:200 dilution) and CD16 (BioLegend, clone: 3G8, 980104, 1:200 dilution). Intracellular staining for proinflammatory cytokines IL-8 (BioLegend, clone: BH0814, 514604, 1:200 dilution), IL-6 (BioLegend, clone: MQ2-13A5, 501112, 1:200 dilution) and IL-12 (BioLegend, clone: C8.6, 508807, 1:300 dilution) were performed using the Cyto-Fast Fix perm buffer set (BioLegend, 426803) as per the manufacturer's instructions. The percent of cytokine-producing neutrophils from TREM1-activated cells treated with morin hydrate and NCI188818 were acquired on an Attune NxT Acoustic Focusing flow cytometry platform and analyzed using FlowJo v10.0. From the functional assays, we determined that NCI 118818 and morin hydrate exhibited relatively high cytotoxicity on human neutrophils. To decrease the cytotoxic effect of NCI 118818, new chemical modifications had to be implemented onto the compound; the resulting novel small molecular inhibitor VJDT was synthesized by LeadGeneLabs, NJ (Supplemental

Figure S2E). Additional details are provided in the patent application. The TREM1 inhibitory effect of VJDT was demonstrated in the reporter cells (Supplemental Figure S2F) and in liver injury experiments (detailed in patent application). Extensive titration studies with these models were carried out to find the optimal concentration of VJDT to be 50 µM for effectively blocking TREM1 activation (detailed in patent application). We used this concentration of VJDT for the functional assay in human neutrophils (Supplemental Figure S2G) and all further *in vitro* and *in vivo* experiments. The graphical summary determined that VJDT exhibited the strongest TREM1 inhibition in comparison to other inhibitors such as NCI 118818 and morin hydrate. Cell viability assays of VJDT were performed on HepG2, B16F10, U87 and U251 cells as described below (Supplemental Figure S2H). Oral administration of VJDT to *Trem1*$^{+/+}$ mice (Supplemental Figure S2I) exhibited limited liver toxicity as shown by minimal infiltration of immune cells (Supplemental Figure S2J). VJDT administration did not alter body weight (Supplemental Figure S2K) nor induce significant elevation in liver functional enzymes (Supplemental Figure S2L).

Cell culture, reagents, and tumor cell lines: The human hepatocellular carcinoma cell line HepG2 was purchased from American Type Culture Collection (ATCC, HB-8065) and was cultured in DMEM media (Corning, 15-013-CV) supplemented with 10% heat-inactivated FBS (Hyclone, SH30396.03), 100 IU/ml of penicillin, and 100 µg/ml of streptomycin (Corning, 30-002-CI). The murine melanoma cell line B16-F10 (ATCC, CRL-6475) was maintained in RPMI media (STEMCELL Technologies, 36750)

supplemented with 10% heat-inactivated FBS and penicillin-streptomycin. The murine fibrosarcoma cell line MCA205 (Millipore, SCC173) was cultured in FBS and penicillin-streptomycin containing RPMI media supplemented with MEM non-essential amino acids (Cellgro, 25-025-CI). TREM1 reporter cells were kindly provided by Prof. Marina Cella, Washington University, St. Louis, MO. The reporter cells were maintained in RPMI supplemented with 10% heat inactivated FBS and non-essential amino acids (Cellgro, 25-025-LI). All cells were maintained in a humidified incubator at 37^0C with 5% CO_2 and were regularly tested to confirm they were free of mycoplasma contamination.

Cell viability assay: Cell viability was determined using the 3-(4,5-dimetiltiazol-2-il)-2,5-difeniltetrazolio (MTT) and the CellTiter-Glo viability (Promega, G7570) assays (Supplemental Figure S2H). B16F10 and HepG2 cells (10^5 cells/well) were plated in 96-well tissue culture plates in complete medium (100μl/well). The multi-well plates were incubated at 37^0C, 5% CO_2 for 24 hrs. After cell adhesion, the culture medium was removed and equal volumes (100μl/well) of the treatments were added to each well. In vehicle control wells, 100μl of DMSO was added. 72 hrs later, proliferative cells were detected by MTT by recording its absorbance at 450nm and via luminescence for the CellTiter assay using a Biotek Synergy multi-well spectrophotometer. The IC_{50} was calculated using GraphPad Prism 9.

Liver toxicity study: Toxicity studies for VJDT treatment with either intraperitoneal or oral administration for 12 days were carried out as described (Supplemental Figure S2I). Liver tissues were fixed in 4% paraformaldehyde and embedded with paraffin. 7 µm sections were stained with hematoxylin and eosinophil (H & E) (Thermo Fisher Scientific). All images were captured by a Keyence BZ-9000 microscope (Keyence). Serum ALT and AST levels were measured with the calorimetric assays Liquid ALT and Liquid AST (Pointe Scientific, 2366087) according to the manufacturer's recommendation.

Wound healing assay: HepG2 or B16F10 cells (3×10^5 cells/well) were seeded in 24-well plates and allowed to grow to monolayers for 72 hrs and 24 hrs, respectively. A sterile 20–200 µL pipette tip was held vertically to scratch a cross in each well. The detached cells were removed by washing with 500 µL PBS. 500 µL of fresh medium with either VJDT (10 µM or 50 µM) or the vehicle (DMSO) were added, and cultures incubated. For HepG2 cells after 24 hrs of treatment, cells were washed with 500 µL PBS, pre-warmed medium added and images collected at 72 hrs. Before image acquisition, the plate was washed with 500 µL PBS before adding pre-warmed medium. The scratch closure was monitored and imaged using a Keyence BZ-9000 microscope (Keyence).

Fluorescent multiplexed immunohistochemistry assay, imaging, and quantitation: Human tumor and corresponding control tissue sections were

purchased from US Biomax. These included paired cancer and normal brain (NCT001), cancer and normal liver (NCT046), cancer and normal breast (NCT121) and cancer and normal skin (NCT246) tissues. The sections were deparaffinized for multiplexed staining using the Opal protocol. Per the manufacturer's instructions, paraffin-embedded tumor tissue specimens were heated at 57°C overnight, residual paraffin was removed using xylene, and tissues were rehydrated in a series of graded alcohols to distilled water. Antigen retrieval was performed according to the manufacturer's protocol (Perkin Elmer). Following cooling and washing with TBST, tissue sections were blocked by 30 min incubation with 10% normal goat serum (Jackson ImmunoResearch, 005-000-121). Subsequent multiplexed staining of the tissue sections was carried out using the manual Opal 7-Color IHC Kit (Akoya-Perkin Elmer, NEL811001KT) per the manufacturer's instructions. Tissue sections were then incubated for 1 hr with primary antibody, followed by incubation with Opal polymer HRP Ms + Rb (as secondary antibody). The sections were washed, and the tyramide signal amplification (TSA)-dye (Akoya-Perkin Elmer, NEL811001KT) was applied. The process was repeated for staining with all primary antibodies: CD11b (Novus Biological, NB11089474, 1:500 dilution), CD68 (BioLegend, clone: BL13756, 375602, 1:700 dilution), and TREM-1 (Miltenyi Biotec, clone: REA213, 130-101-040, 1:200 dilution). Nuclei were stained with ProLong™ Gold antifade reagent with DAPI (Invitrogen, P36931). Multiplexed imaging was performed with Opal 7-Color Manual IHC Kit, and the multiplexed sections were imaged at x20 on the Vectra Automated Quantitative Pathology Imaging System. Quantitation of chromogenic, fluorescent

signals and percent of positive/negative cells for each marker was performed using InForm v2.3.0 (Perkin Elmer).

Cell proliferation assay: The five stably transfected HepG2-*shTREM1* cell lines and the shControl clones were assayed for cell proliferation with the WST-1 reagent (Sigma-Aldrich, 05015944001) per kit instructions. Briefly, HepG2 cells ($4x10^3$ cells/well in 200 µl culture medium) were seeded into a 96-well flat-bottom tissue culture plate. Cell proliferation reagent WST-1 (Sigma, 5015944001, 20 µl/well) was added, and cells were incubated at 37°C in a humidified CO_2 incubator. Absorbance was measured repeatedly using a microplate (ELISA) reader at 440 nm wavelength and reference wavelength at 600 nm. The first absorbance was taken 4 hours after incubation and counted as day 0; the subsequent measurements were taken every 24 hours until day 5.

Cell cycle analysis: 10^6 of the stably transfected HepG2-*shTREM1* cells and the shControl clones were seeded onto a 6-well plate containing the appropriate culture media. Each clone was treated with VJDT at 10µg or 50µg or DMSO for 24 hours. The cells were harvested by trypsinization, washed twice with PBS, and fixed in ice-cold 70% ethanol. The cells were washed again, subjected to RNase A at a final concentration of 100µg/ml (Qiagen, 19101) and incubated with 50µg/ml propidium iodide (BioLegend, 421301) at 4°C for 15 min in the dark. Samples were acquired on a FACSCanto (BD Biosciences) platform, and cell cycle analysis was performed with FlowJo v10.0.

RNA isolation and reverse transcription-quantitative PCR (RT-qPCR): Total RNA was isolated from tumor specimens and the transfected HepG2 cell cultures using TRIzol reagent (ThermoFisher Scientific, 15-596-026) followed by purification with the RNeasy Mini kit (Qiagen, 74004) per the manufacturers' instructions. Total RNA concentration was measured by NanoDrop spectrophotometer (ThermoFisher Scientific, ND-2000). cDNA was synthesized from 750 ng of total RNA using RT^2 First Strand kit (Qiagen, 330401) per the manufacturer's instructions. RT-qPCR mixes were set up using SYBR Green qPCR Master Mix (Bimake, B21203), and the reactions were carried out in duplicate in a StepOnePlus™ real-time PCR system (Applied Biosystems, 4376600) at 95°C (3 min) followed by 40 cycles of 95°C (15 sec), 64°C (45 sec). Fold change was determined by calculating the ratio of mRNA levels to control values using the Δ threshold cycle (Ct) method ($2^{-\Delta\Delta Ct}$). All data were normalized based on the average of three housekeeping genes, *ACTB*, *GAPDH*, and *HPRT1,* and the values are expressed as fold induction in comparison to the analyzed group. Gene expression analysis was performed using specific sets of primers for *CDC20* (forward 5´-GGCACCAGTGATCGACACATTCGCAT-3´, reverse 5´-GCCATAGCCTCAGGGTCTCATCTGCT-3´)(2); *IL1B* (forward 5´-CCACAGACCTTCCAGGAGAATG-3´, reverse 5´-GTGCAGTTCAGTGATCGTACAGG-3´); *CXCL10* (forward 5´-GGTGAGAAGAGATGTCTGAATCC-3´, reverse 5´-GTCCATCCTTGGAAGCACTGCA-3´)(3); *CXCL8* (forward 5´-GAGAGTGATTGAGAGTGGACCAC-3´, reverse 5´-

CACAACCCTCTGCACCCAGTTT-3´)(4); *NFKB1* (forward 5´-GCAGCACTACTTCTTGACCACC-3´, reverse 5´- TCTGCTCCTGAGCATTGACGTC-3´); *STAT3* (forward 5´- CTTTGAGACCGAGGTGTATCACC-3´, reverse 5´-GGTCAGCATGTTGTACCACAGG-3´); *JUN* (forward 5´-CCTTGAAAGCTCAGAACTCGGAG-3´, reverse 5´-TGCTGCGTTAGCATGAGTTGGC-3´); and *IL6* (forward 5´-AGACAGCCACTCACCTCTTCAG-3´, reverse 5´- TTCTGCCAGTGCCTCTTTGCTG-3´).

Microarray gene expression profiling: Freshly harvested tumors from the PDX or sh*TREM1* HepG2 xenografts were processed into single-cell suspensions as described previously, and RNA purity and concentration were evaluated by spectrophotometry using a NanoDrop spectrophotometer (ThermoFisher Scientific). RNA quality was assessed by the Agilent 2200 TapeStation (Agilent Technologies, G2964AA) and assured of an RNA Integrity Number (RIN) ≥ 7. Total RNA samples were processed using the GeneChip WT PLUS Reagent Kit (ThermoFisher Scientific, 902280). 250 ng of total RNA was used to generate sense-strand cDNAs. The synthesized sense-strand cDNAs (5.5 µg) were fragmented and biotin-labeled according to the manufacturer’s protocol. The labeled samples were then hybridized to the Clariom S human array (ThermoFisher Scientific, 902927) that contains more than 22,100 well-annotated genes and 150,300 transcripts. After 16 hours of hybridization, the arrays were washed and stained using Affymetrix GeneChip Fluidics

Station 450. The stained arrays were scanned on an Affymetrix GeneChip Scanner 3000. Data were obtained in the form of a CEL file. The CEL files were imported into Partek Genomic Suites v6.6 (Partek) using the standard import tool with a Robust Multichip Average (RMA) normalization. The differential expression was calculated using ANOVA of Partek package and filtered with a *p-value* cutoff of 0.05 and fold-change cutoffs to screen out the differentially expressed genes in each comparison. The significant gene list was used to generate a hierarchical clustering plot by the standardized expression values.

Supplemental References

1. Trott O, and Olson AJ. AutoDock Vina: improving the speed and accuracy of docking with a new scoring function, efficient optimization, and multithreading. *J Comput Chem.* 2010;31(2):455-61.
2. Chang DZ, Ma Y, Ji B, Liu Y, Hwu P, Abbruzzese JL, et al. Increased CDC20 expression is associated with pancreatic ductal adenocarcinoma differentiation and progression. *Journal of hematology & oncology.* 2012;5(1):15.
3. Kim HJ, Ryu H, Song J-Y, Hwang S-G, Jalde SS, Choi H-K, et al. Discovery of Oxazol-2-amine Derivatives as Potent Novel FLT3 Inhibitors. *Molecules.* 2020;25(21):5154.
4. Yu X, Liu W, Fan Z, Qian F, Zhang D, Han Y, et al. c-Myb knockdown increases the neomycin-induced damage to hair-cell-like HEI-OC1 cells in vitro. *Sci Rep.* 2017;7:41094.

Supplemental Figure S1. Ajith et al.

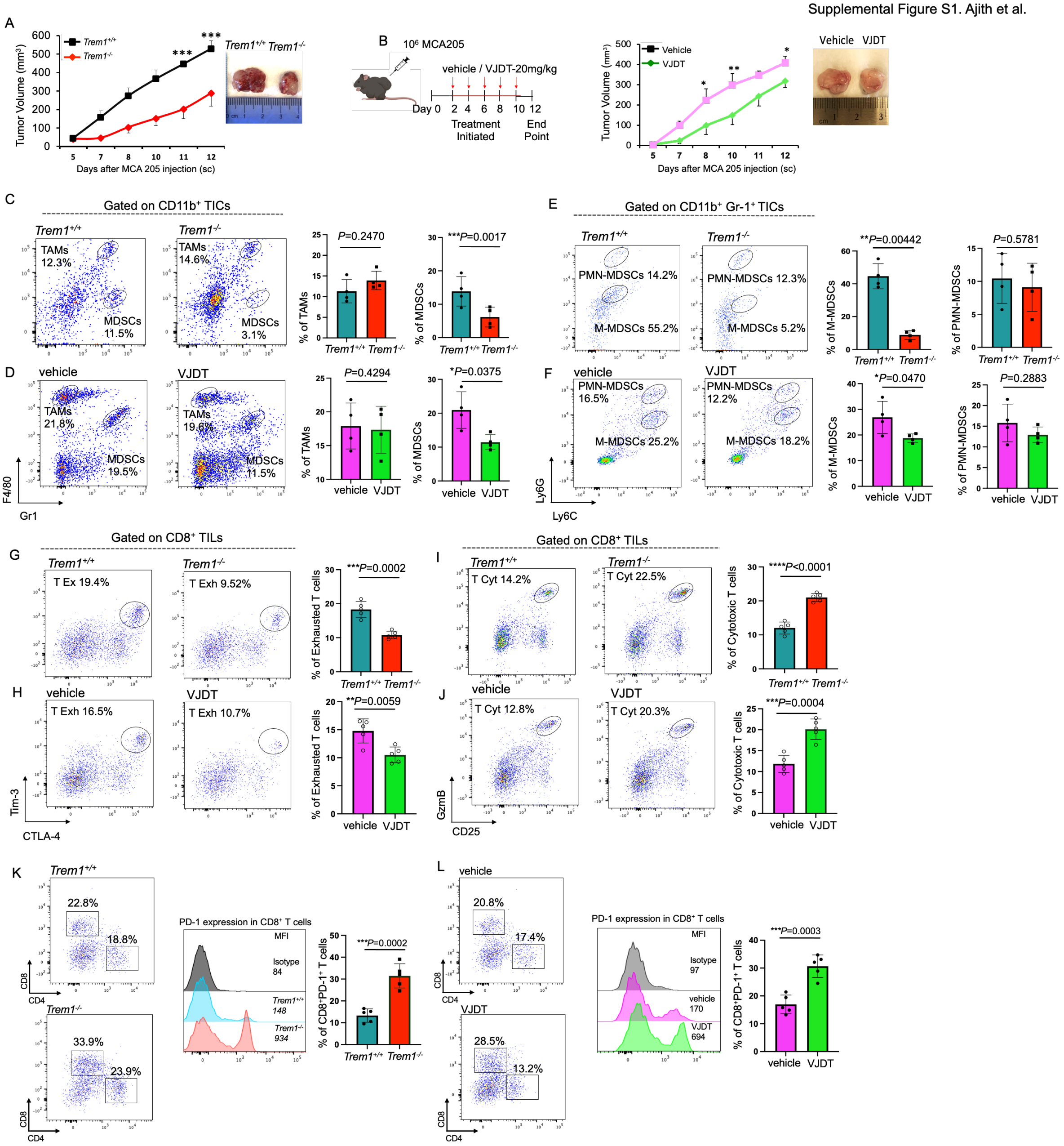

Supplemental Figure S1. TREM1 deficiency and anti-TREM1 treatment diminish MCA205 tumor growth by altering tumor immune infiltrates. (A) Tumor growth curves for *Trem1*$^{+/+}$ and *Trem1*$^{-/-}$ mice with MCA205 fibrosarcoma (n=9 mice/group,

mean ± SEM). Representative microscopic images of the tumors from the indicated groups at day 12 are shown. **(B)** Schematic of treatment with TREM1 inhibitor VJDT on MCA205 fibrosarcoma in *Trem1*$^{+/+}$ mice. Treatment was initiated on 2nd day of tumor growth followed every alternate day with either VJDT (20mg/kg) or vehicle (DMSO) injection until day 10. Tumor growth curves were calculated by individual measurements and recorded every alternate day (n=9 mice/group, mean ± SEM). Representative microscopic images of the tumors from the indicated groups at day 12 are shown. (**C-J**) Tumors were harvested on day 12 and flow cytometry performed (plots depict 1 representative experiment done in triplicate, n=4-5 mice/group, mean ± SEM shown). (**C**) Frequency of CD11b^{+}F4/80^{+}Gr1^{-} TAMs, and CD11b^{+}F4/80^{-}Gr1^{+} MDSC infiltrates in *Trem1*$^{+/+}$ or *Trem1*$^{-/-}$ mice and (**D**) in *Trem1*$^{+/+}$ mice with indicated treatment. (**E**) Frequency of CD11b^{+}Gr1^{+}Ly6C^{high}Ly6G^{-} M-MDSCs and CD11b^{+}Gr1^{+}Ly6C^{low}Ly6G^{+} PMN-MDSCs in *Trem1*$^{+/+}$ or *Trem1*$^{-/-}$ mice and (**F**) in *Trem1*$^{+/+}$ mice with indicated treatment. (**G**) Frequency of exhausted CD8^{+}Tim-3^{+}CLA-4^{+} T cells in *Trem1*$^{+/+}$ or *Trem1*$^{-/-}$ mice and (**H**) in *Trem1*$^{+/+}$ mice with indicated treatment. (**I**) Frequency of cytotoxic CD8^{+}GzmB^{+}CD25^{+} T cells in *Trem1*$^{+/+}$ or *Trem1*$^{-/-}$ mice and (**J**) in *Trem1*$^{+/+}$ mice with indicated treatment. (**K**) PD-1 expression in tumor-infiltrating CD8^{+} and CD4^{+} T cells of *Trem1*$^{+/+}$ or *Trem1*$^{-/-}$ mice and (**L**) in *Trem1*$^{+/+}$ mice receiving indicated treatment (n=5 mice/group, mean ± SEM), assessed by flow cytometry. *P<0.05; **P<0.01; ***P<0.001; ****P<0.0001 by two-way ANOVA for multiple comparison of longitudinal tumor growth between various groups (**A,B** [tumor growth]) or two-tailed Student's *t*-test for comparison between 2 groups (**C-L**).

Supplemental Figure S2. Ajith et al.

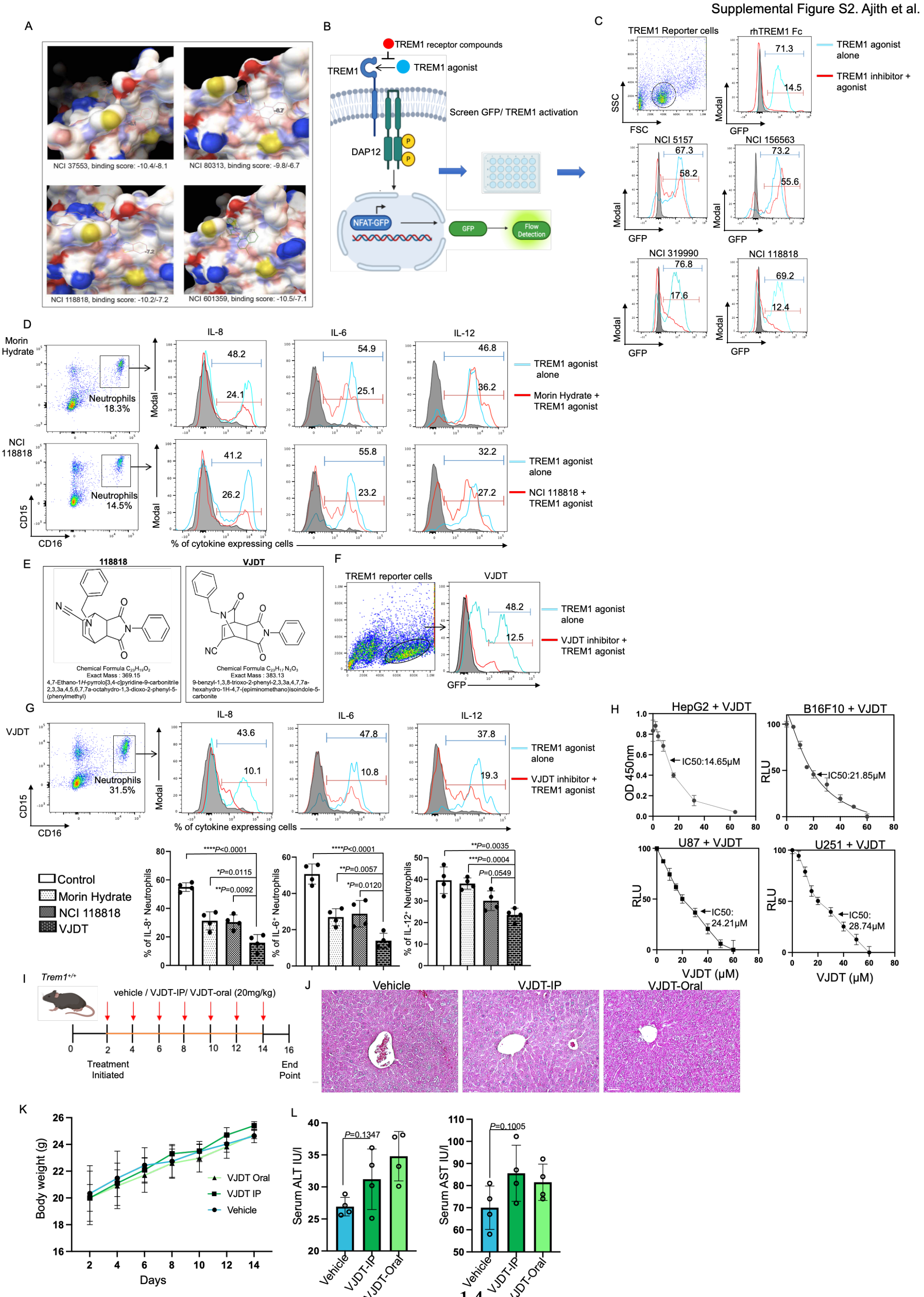

Supplemental Figure S2. Development of novel TREM1 inhibitor VJDT. (**A**) Autodock Vina 1.1.12 was used for receptor-based virtual screening of the NCI diversity database containing 1,593 candidate drugs against TREM1 receptor. Computational molecular docking was used to predict binding score and select the top candidates blocking TREM1. (**B**) Illustration shows screening of potential TREM1 inhibitors. TREM1 responsive reporter cell line expresses TREM1 from an NFAT response element-driven transgene. A GFP reporter was introduced into the cells to monitor TREM1 transcriptional activity. (**C**) Flow cytometry analysis depicts percentage of GFP activation by TREM1 binding in reporter cells treated with the indicated compounds. (**D**) Flow cytometry histograms depict cytokine expression in human neutrophils during stimulation with TREM1 agonist alone (blue line) and during TREM1 inhibition with blocking compound (morin hydrate, NCI 118818) followed by stimulation with the agonist. (**E**) Chemical structures of 118818 (4,7-Ethano-1H-pyrrolo[3,4-c]pyridine-9-carbonitrile2,3,3a,4,5,6,7,7a-octahydro-1,3-dioxo-2-phenyl-5-(phenylmethyl)), a synthetic compound from the NCI diversity database, and the newly synthesized TREM1 inhibitor VJDT ((3aR,4R,7R)-9-benzyl-1,3,8-trioxo-2-phenyl-2,3,3a,4,7,7a-hexahydro-1H-4,7-(epiminomethano)isoindole-5-carbonite). (**F-G**) Flow cytometry histograms depict TREM1 activation in (**F**) reporter cells and (**G**) neutrophils with the agonist AF1278 (4μg/ml for 24 h) alone in blue, and in combination with VJDT pre-incubation (50μM for 6 h) followed by stimulation with AF1278 in red. Data representative of 4 independent experiments (mean $\pm$ SEM). (**H**) IC_{50} of VJDT in HepG2 B16F10, U251 and U87 cells calculated by MTT and the CellTiter-Glo viability

assay. (**I**) Illustration of vehicle (DMSO and IgG), VJDT intraperitoneal or oral (20mg/kg) treatment on *Trem1*$^{+/+}$ mice for toxicity assessment (n=4 mice/group, mean ± SEM). (**J**) After 12 days of VJDT intraperitoneal or oral treatment, histological architecture of liver was evaluated using H & E staining. Original magnification, 20X; scale, 50μm. (**K**) Mouse body weight curves in the treatment groups across 12 days. (**L**) Hepatic functional indicators ALT and AST assessment in the treatment groups. *P<0.05; **P<0.01; ***P<0.001; ****P<0.0001 by two-tailed Student's t-test for comparison between 2 groups (**G-L**).

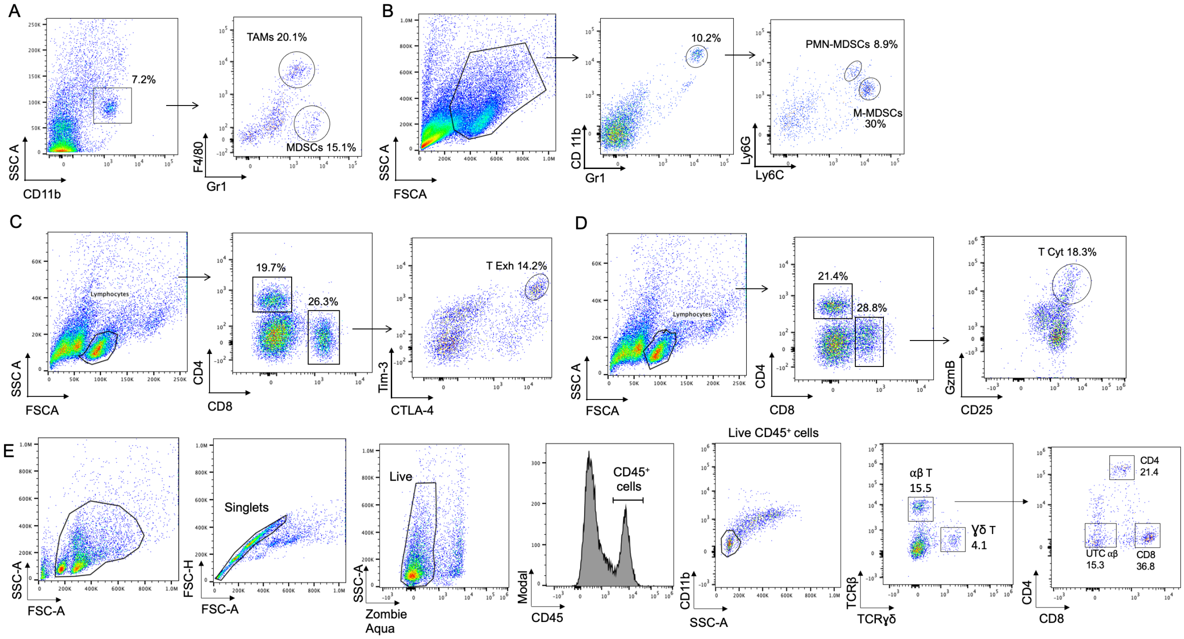

Supplemental Figure S3. Representative flow cytometry gating strategy for characterization of tumor infiltrating cells within the TME. Related to Figures 1, 5 and 7. (**A-D**) Dot plots depict gating strategy for identification of (**A**) TAMs and MDSCs. (**B**) PMN-MDSCs and M-MDSCs, (**C**) Exhausted T cells and (**D**) Cytotoxic T cells. (**E**) Flow cytometry gating strategy characterizing the $UTC_{\alpha\beta}$ cells in the TME, related to Figure 7.

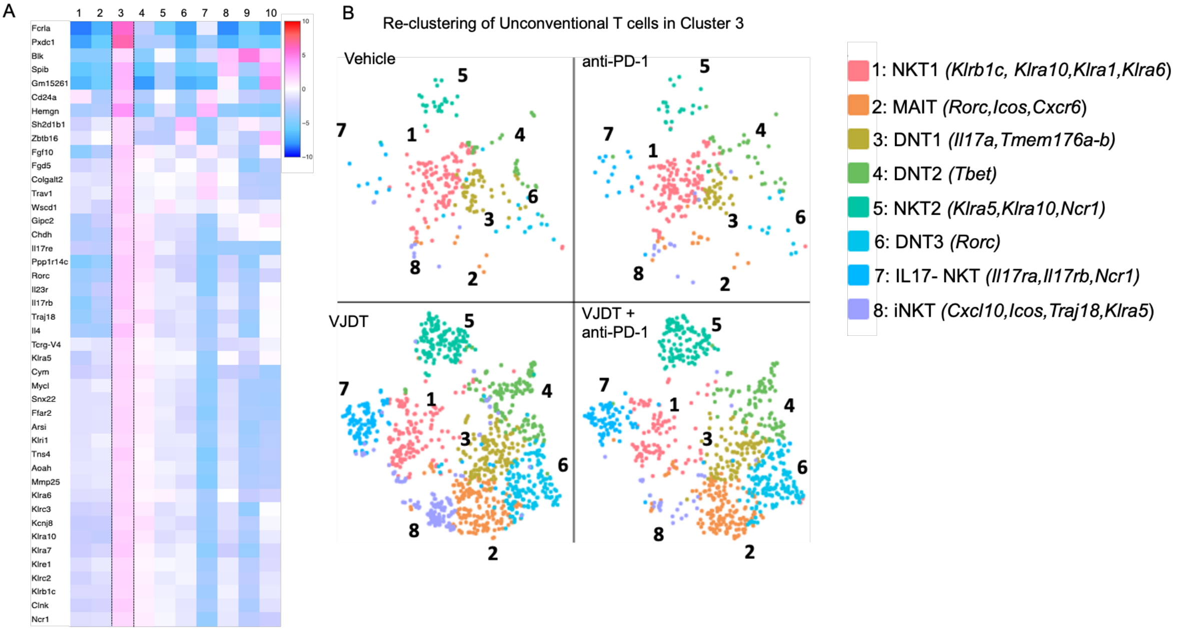

Supplemental Figure S4. TREM1 inhibition leads to altered polarization within tumor infiltrating UTC$_{\alpha\beta}$ subsets. Related to Figure 7. (**A**) Heatmap depicts differential expression of specific genes expressed by UTC$_{\alpha\beta}$ subset across all clusters in our scRNA-seq analysis. Cluster 3 exclusively expressed 47 of the top 50 markers associated with UTC$_{\alpha\beta}$ cells. (**B**) t-SNE plot describes re-clustering analysis of unconventional T cells identified in the scRNA-seq data. Cluster identities were annotated based on key gene signatures as described.

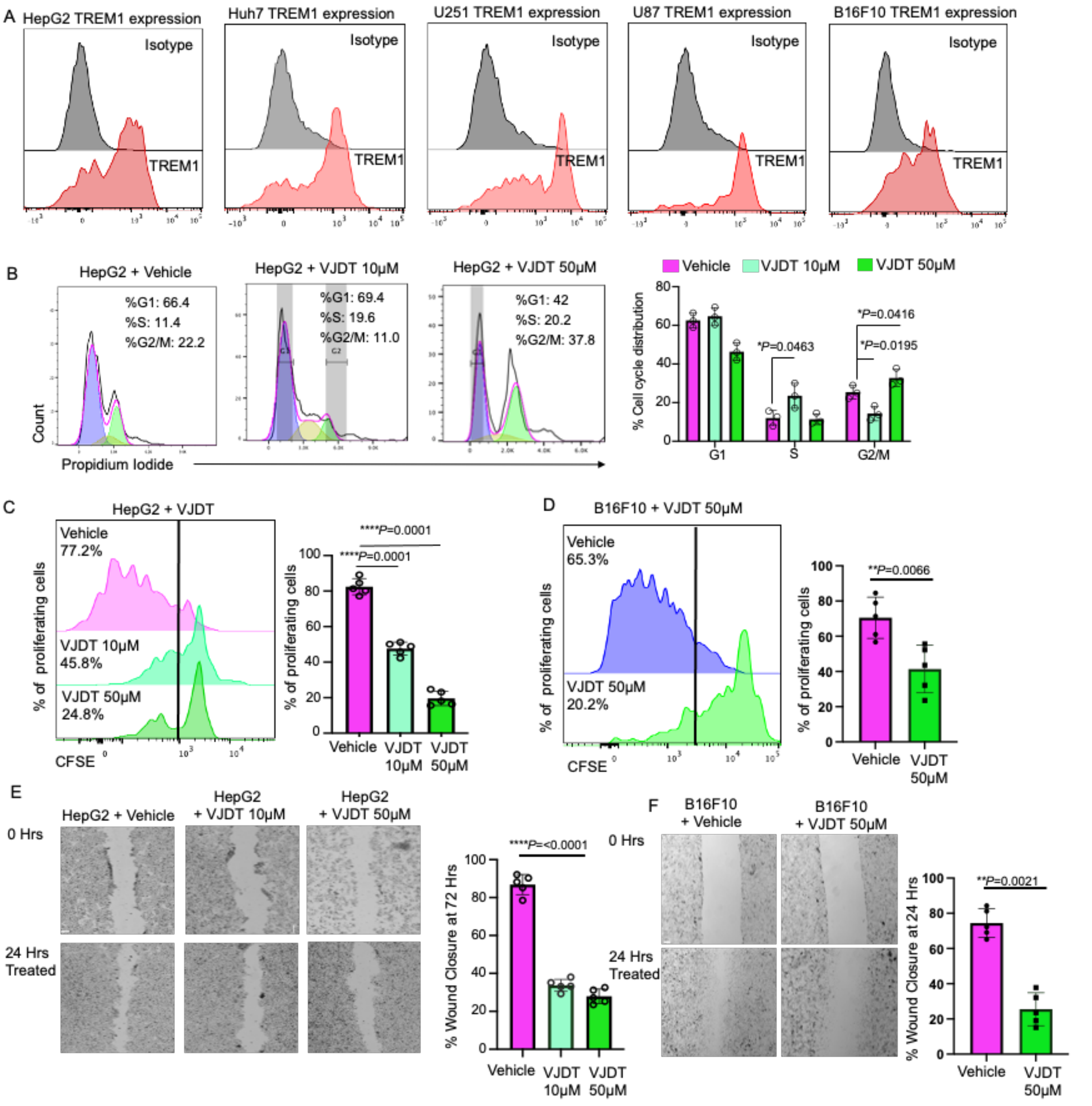

Supplemental Figure S5. VJDT treatment inhibits B16F10 and HepG2 cell proliferation and migration. Related to Figure 8. (**A**) Representative flow cytometry histograms depict TREM1 expression in various cancer cells. (**B**) Flow cytometry

histograms of cell cycle analysis and quantification of the cells in G0/G1, S and G2/M phases in HepG2 cells treated with VJDT (10μM or 50μM) or vehicle for 24 h. Data are representative of 3 independent experiments (n=3/group, means ± SEM). (**C-D**) Representative flow cytometry histograms and quantification of proliferating cells in CFSE-labeled (**C**) HepG2 or (**D**) B16F10 cells treated with VJDT (10μM or 50μM) or vehicle (DMSO) for 24 h. Data are representative of 5 independent experiments (n=5/group, means ± SEM). (**E-F**) Wound healing assay of (**E**) HepG2 and (**F**) B16F10 cells treated with VJDT or vehicle (DMSO) for 24 h. Representative images of HepG2 cells at 72 h and B16F10 cells at 24 h are shown. Scale Bar, 200μm. Data are representative of 5 independent experiments (n=5/group, means ± SEM). *P<0.05; **P<0.01; ****P<0.0001 by two-tailed Student's t-test for comparison between two groups (**A-F**).

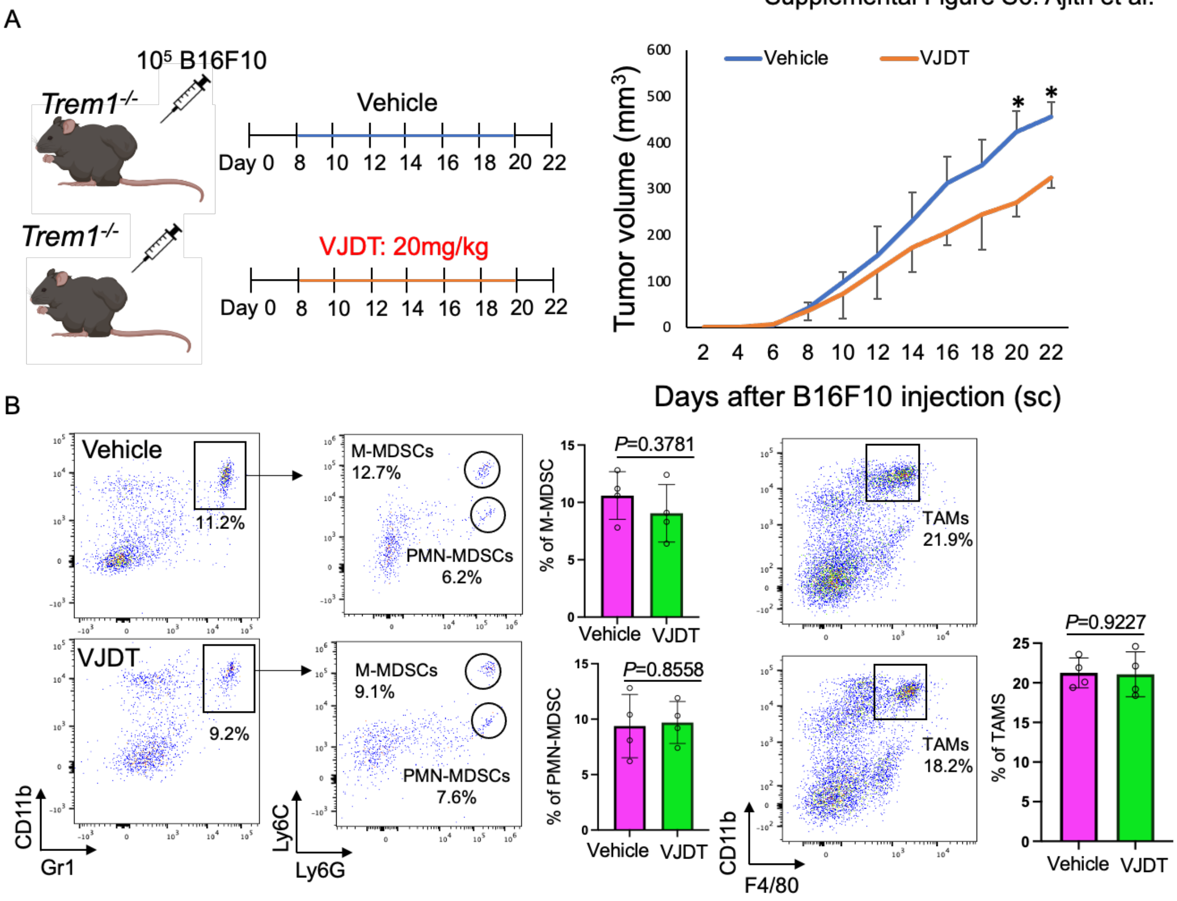

Supplemental Figure S6. VJDT treatment restrains B16F10 tumor growth in *Trem1$^{-/-}$* mice. (**A**) Illustration shows VJDT treatment protocol on B16F10 tumor growth. VJDT (20mg/kg) or vehicle (DMSO) treatment was initiated 8 days after B16F10 cell injection into *Trem1$^{-/-}$* mice and continued every alternate day until day 22. Tumor growth curve of B16F10 melanoma after treatment with VJDT or vehicle are shown. Data are represented as means ± SEM, n=4 mice per group. *P* values from two-way ANOVA are reported, **P*<0.05. (**B**) Representative flow cytometry dot plots depict gating strategy for the identification of M-MDSCs, PMN-MDSCs and TAMs in

immune infiltrates from *Trem1*$^{-/-}$ mice tumors at day 22. The box plots describe quantification of TICs in each group. Data are represented as means ± SEM, n=4 mice per group. *P* value calculated by two-tailed Student's *t*-test for comparison between two groups.
